# Association of Childhood Maltreatment With Suicide Behaviors Among Young People: A Systematic Review and Meta-analysis

**DOI:** 10.1001/jamanetworkopen.2020.12563

**Published:** 2020-08-05

**Authors:** Ioannis Angelakis, Jennifer L. Austin, Patricia Gooding

**Affiliations:** 1 School of Psychology, University of South Wales, Pontypridd, United Kingdom; 2 Division of Psychology and Mental Health, School of Health Sciences, Faculty of Biological, Medical and Health Sciences, University of Manchester, Manchester, United Kingdom; 3 Manchester Academic Health Science Centre (MAHSC), Manchester, United Kingdom

## Abstract

**Importance:**

Children and young people’s reports of experiences of adverse childhood events have increased in recent years, and this trend has been associated with an elevated risk for suicide behaviors. However, a systematic review and meta-analysis is needed to confirm the significance of this association in young people.

**Objective:**

To quantify the association between core types of childhood maltreatment, including sexual, physical, and emotional abuse and/or neglect and suicide behaviors in children and young adults.

**Data Sources:**

Medline, PsychInfo, Embase, Web of Science, and CINAHL (Cumulative Index to Nursing and Allied Health) databases were searched from January 1, 1980, until December 31, 2019. The reference lists of all the included studies were also checked.

**Study Selection:**

Quantitative studies that focused on the association between core types of childhood abuse and/or neglect and suicide ideation, plans, and attempts.

**Data Extraction and Synthesis:**

Data were extracted by 2 independent raters. Publication bias and risk of bias across studies were assessed. Meta-analyses using random-effect models were applied, and heterogeneity was quantified using the *I*^2^ statistic. Data were analyzed from January to May 2020 in accordance with the Preferred Reporting Items for Systematic Reviews and Meta-analyses (PRISMA) and Meta-analysis of Observational Studies in Epidemiology (MOOSE) reporting guidelines.

**Main Outcomes and Measures:**

The association between core types of childhood maltreatment and suicide behaviors.

**Results:**

Seventy-nine studies with 337 185 young individuals (mean [SD] age, 15.67 [2.11] years; 63.19% female) were included. The findings demonstrated that sexual abuse (odds ratio [OR], 3.41; 95% CI, 2.90-4.00), physical abuse (OR, 2.18; 95% CI, 1.75-2.71), emotional abuse (OR, 2.21; 95% CI, 1.37-3.57), emotional neglect (OR, 1.93; 95% CI, 1.36-2.74), physical neglect (OR, 1.79; 95% CI, 1.27-2.53), and combined abuse (OR, 3.38; 95% CI, 2.09-5.47) were significantly associated with higher rates of suicide attempts. Core types of childhood maltreatment were also associated with as much as 2.5-fold greater odds for suicide ideation, and sexual abuse with a 4.0-fold increase for suicide plans. Studies based on community samples (β [SE] = −1.68 [0.79]; *P* = .04) or with lower methodological quality (β [SE] = −2.86 [1.30]; *P* = .03) were associated more strongly with suicide attempts in those reporting experiences of sexual abuse, whereas young age was associated with both suicide attempts (β [SE] = −0.59 [0.27]; *P* = .03) and ideation (β [SE] = −0.41 [0.18]; *P* = .03).

**Conclusions and Relevance:**

These findings suggest that policy actions should focus on raising public awareness and offering proactive suicide prevention therapies for children and young adults who have experienced abuse and/or neglect.

## Key Points

**Question:**

What is the association between experiences of childhood maltreatment and suicide behaviors in children and young adults?

**Findings:**

This systematic review and meta-analysis was based on 79 individual studies with 337 185 unique participants found an association between core types of childhood maltreatment and suicide behaviors in children and young adults. Younger individuals with experiences of sexual abuse who were not under the care of clinicians had higher rates of suicide attempt, and young age was also associated more strongly with suicide ideation.

**Meaning:**

These findings highlight the need for raising public awareness and incorporating suicide prevention strategies into treatment planning and suggest that a primary focus of psychological treatments should be the amelioration of the effects of adverse childhood experiences.

## Teaser

This systematic review and meta-analysis assesses the association between experiences of childhood maltreatment and suicide behaviors in children and young adults.

## Introduction

Childhood maltreatment constitutes experiences of any sexual, physical, and emotional abuse and/or neglect that result in substantiated or possible harm that affects the individual’s physical and mental health.^1^ The rate of maltreatment experienced by 18 years of age was estimated to be 12.5% in a representative US sample.^2^ In the United Kingdom, the rate of maltreatment of children and adolescents aged 11 to 17 years was 18.6%.^1^

Increasing evidence suggests that childhood maltreatment is strongly associated with self-harm, suicide behavior, lower resilience to mental health problems, and greater impulsivity.^3,4,5,6,7^ The severe negative psychological consequences of experiencing childhood abuse and/or neglect often continue into adulthood in the form of substantial mental health problems, including depression, anxiety, and posttraumatic stress disorder.^8,9^ In addition, these individuals may engage in behaviors that negatively affect their health, including risky sexual behavior^10^ or using drugs and/or alcohol.^11^ Hence, experiences of child maltreatment significantly contribute to societal costs by increasing the mental and physical health care provision needs for those who have experienced abuse and neglect.^12^

The rates of deaths due to suicide exceed 800 000 people each year globally.^13^ This means that almost 1 individual takes his or her own life every 40 seconds. Suicide is the second leading cause of death among young people aged 15 to 24 years, and the rates of young people losing their lives to suicide has grown in recent years.^14^ Recent evidence suggests that the number of hospitalizations of those attempting to take their own lives has doubled within the past decade, with suicide attempts considerably higher in children and adolescents aged 12 to 17 years.^15^

To date, 5 reviews have examined the association between childhood maltreatment and suicide behavior in children and young adults.^16,17,18,19,20^ Two key limitations of these reviews include lack of meta-analyses to quantify the existing evidence, especially for suicide behaviors other than attempts (ie, suicide ideation and plans), and restrictive inclusion criteria for specific research designs, leading to the exclusion of a considerable number of studies conducted in this area. Furthermore, scant evidence is available regarding the influence of key methodological factors and sample characteristics on this association. Given that recent evidence suggests both an increase in the recorded adverse events in childhood and an increase in suicide attempts in children and young people,^2,15^ we undertook, to our knowledge, the first comprehensive systematic review and meta-analysis to bridge this research gap regarding the association between childhood maltreatment and the various suicide behaviors in youth. The study had 3 key objectives:

1. To systematically quantify the association between core forms of childhood maltreatment and suicide attempts in samples of children and young adults to 24 years of age;
2. To evaluate, whenever possible, the strength of this association across the different modes of suicide behavior, including suicide ideation and plans; and
3. To explore key study factors (eg, methodological quality) and sample-related characteristics (eg, age, sex, type of population) that may affect the strength of the association between experiences of abuse/neglect and suicide behaviors in youth.

## Methods

This systematic review and meta-analysis was prepared and conducted in accordance with the Preferred Reporting Items for Systematic Reviews and Meta-analyses (PRISMA)^21^ statement and the Meta-analysis of Observational Studies in Epidemiology (MOOSE) reporting guideline.^22^

### Inclusion and Exclusion Criteria

All the studies that were included in the review met the following eligibility criteria:

1. Included participants aged 5 to 24 years^23^ who had experienced any form of abuse and/or neglect before 18 years of age;
2. Used quantitative research designs;
3. Reported quantitative outcomes of the association between core forms of childhood maltreatment and suicide experiences, including suicide thoughts, plans, and/or attempts; and
4. Were published in peer-reviewed journals in English.

Qualitative studies, case series, case studies, position papers, reviews, dissertations, theses, articles that focused on other forms of childhood adversities, such as witnessing violence and parental deaths or divorces, and those that did not provide data appropriate for meta-analyses (eg, reported data on suicide acts and experiences of abuse separately) were omitted.

### Search Strategy and Data Sources

Five electronic bibliographic databases were searched, including Medline, PsychInfo, Embase, Web of Science, and CINAHL (Cumulative Index to Nursing and Allied Health). The reference lists of the identified studies were also searched. We also contacted authors for additional information when necessary.^24^ The searches were performed from January 1, 1980, until December 31, 2019. Searches included both text words and MeSH (Medical Subject Headings) terms and combined 3 blocks of key terms: (1) suicide (suicid* OR suicide* correl* OR self*harm), (2) child/sexual/physical/emotional abuse or neglect or maltreatment or adversities (child*, sex*, phys*, emoti* abuse, negl*, maltreat*, advers*), and (3) adolescents (adolesc*, youth*, teenager, kid, boy, girl).

### Study Selection and Data Extraction

The titles, abstracts, and the full texts of the identified studies were scrutinized by 2 independent reviewers (I.A. and J.L.A.). We assessed interrater reliability for title and abstract screening (κ = 0.92) and for full-text screening (κ = 0.93), both of which were high. We extracted descriptive information, including participant characteristics (eg, age, sex), study characteristics (eg, country, methodological design, method of recruitment), screening tools for childhood maltreatment and suicide experiences, forms of childhood maltreatment (eg, sexual, physical, and emotional or psychological abuse and emotional or physical neglect), modes of suicide experiences (eg, ideation, plans, and attempts), and type of sample (eg, community samples with or without formal psychiatric diagnoses, psychiatric inpatients). Interrater agreement was excellent (κ = 0.94). Disagreements were resolved by discussion.

### Appraisal of Methodological Quality

Similar to other studies published in this area,^3^ criteria based on the Centre for Reviews and Dissemination guidance^25^ were used to assess the methodological quality of the papers. These criteria included (1) research design (1 indicates cross-sectional; 2, prospective or experimental), (2) baseline response rate (1 indicates ≤70% or not reported; 2, ≥70%), (3) follow-up response rate (1 indicates ≤70% or not reported; 2, ≥70%), (4) screening tools for childhood adversities (1 indicates not reported or self-report scale; 2, structured or semistructured clinical interview), (5) screening tools for suicidality (1 indicates not reported or self-report scale; 2, structured or semistructured clinical interview), and (6) control for confounding or other factors in the analysis (1 indicates not controlled or not reported; 2, controlled). Studies that scored 3 or lower were considered to be low-quality studies.^26^ These scores were also entered into the multivariate meta-regression models to perform sensitivity analyses.

### Statistical Analysis

Data were analyzed from January to May 2020. All meta-analyses were conducted in STATA, version 15 (StataCorp LLC). Odds ratios (ORs) were calculated as the preferred effect size because most of the studies (n = 65) reported dichotomous outcomes. For those studies (n = 14) that reported continuous data, we used the Comprehensive Meta-analysis program, version 3,^27^ to produce ORs. To avoid double counting of studies in the same analysis, we first grouped all effect sizes according to the distinct forms of childhood maltreatment (eg, sexual, physical, and emotional abuse and physical and emotional neglect) separately. We then classified these effect sizes into individual categories according to the mode of suicide behavior (eg, ideation, plans, and attempts). We performed meta-analyses to assess the pooled effect size of each of the distinct comparison groups using the *metan* command.^28^ We conducted multivariate meta-regression analyses by using the *metareg* command^29^ for those categories that provided a sufficient number of studies (eg, ≥20)^30^ to warrant such an analysis. Meta-regression analyses served to further evaluate the role of the sample (eg, age, sex, and type of population) and study-level moderators (eg, type of research design, screening tools for measuring childhood maltreatment and suicide experiences, and methodological appraisal) in the association between childhood maltreatment and suicide experiences. All meta-analyses were conducted using a random-effects model because substantial heterogeneity (ie, variation in the study outcomes across the studies), which was assessed with the *I*^2^ statistic (ie, the percentage of variation across studies due to heterogeneity), was anticipated across the studies.^31^ Conventionally, a value of 25% denotes low heterogeneity; 50%, moderate heterogeneity; and 75%, high heterogeneity. Provided that each individual comparison group contained more than 9 independent effect sizes, we (1) explored publication bias by producing funnel plots and by examining the significance of the Egger tests^32^ and (2) ran leave-one-out sensitivity analyses to evaluate the robustness of the findings. The trim and fill method of Duval and Tweedie,^33^ which is a method that yields an estimate of the number of missing studies, was used to correct the pooled effect sizes in the case of publication bias. Two-sided *P* < .05 indicated significance.

## Results

Initial searches generated a total of 1607 articles, and 213 duplicates were omitted. Of the remaining 1394 articles, 987 were also omitted because they did not meet all the inclusion criteria. In total, full-text copies of 407 articles (25.33%) were accessed. However, an additional 328 studies were excluded because they did not meet the inclusion criteria for this review. This process left 79 individual studies eligible for inclusion^24,34,35,36,37,38,39,40,41,42,43,44,45,46,47,48,49,50,51,52,53,54,55,56,57,58,59,60,61,62,63,64,65,66,67,68,69,70,71,72,73,74,75,76,77,78,79,80,81,82,83,84,85,86,87,88,89,90,91,92,93,94,95,96,97,98,99,100,101,102,103,104,105,106,107,108,109,110,111^ based on 337 185 unique young individuals (1 study^34^ contained a mixed sample with adults older than 24 years; this study was excluded from the overall sample calculation) (Figure and eTable in the Supplement). The mean (SD) age of the participants was 15.67 (2.11) years, with those identifying as female constituting most of the sample (63.19% vs 36.81% male). Most of the studies were conducted in North America (43 [54.4%]), followed by China or other Asian countries (16 [20.3%]), Europe (11 [13.9%]), Australasia (7 [8.9%]), and South America (1 [1.3%]). A single study (1.3%) reported data that had been recorded across several countries.^34^ Most of the studies (63 [79.7%]) had a low methodological quality (eTable in the Supplement).

**Figure.**
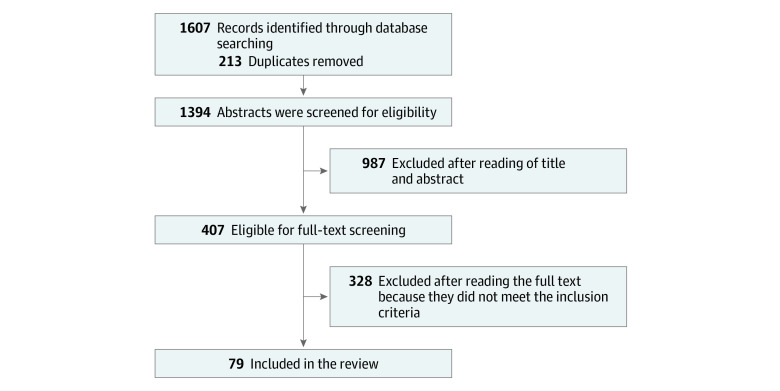
PRISMA Flow Diagram for the Entire Review PRISMA indicates Preferred Reporting Items for Systematic Reviews and Meta-analyses.

### Main Meta-analyses

The pooled ORs for each of the individual types of childhood maltreatment are presented in Table 1 (for forest plots, see eFigures 1-11 in the Supplement). Sexual abuse was associated with 3.5-fold increased odds for suicide attempts (48 studies^24,34,37,38,40,41,43,44,46,47,49,50,51,52,53,55,56,57,61,62,63,64,68,73,74,76,77,81,82,84,85,86,87,88,89,90,93,94,95,97,100,101,102,103,104,106,108,111^; OR, 3.42; 95% CI, 2.90-4.00; *I*^2^ = 97.4%), but heterogeneity was high (funnel plots in eFigure 12 in the Supplement). Physical abuse was associated with a 2-fold increase in the odds for suicide attempts (26 studies^34,37,40,46,47,51,52,54,55,56,57,63,68,70,73,81,85,88,90,91,93,95,98,103,104,111^; OR, 2.18; 95% CI, 1.75-2.71; *I*^2^ = 90.2%). Heterogeneity was high, and there was an indication of publication bias. The trim and fill method was applied to correct parameter estimates for publication bias, and as a result the pooled OR increased to 3.07 (95% CI, 2.95-3.19). Emotional abuse was associated with 2-fold increased odds for suicide attempts (6 studies^57,68,73,88,104,111^; OR, 2.21; 95% CI, 1.37-3.57; *I*^2^ = 95.6%), but the overall number of comparison studies was low. Emotional neglect was associated with increased odds for suicide attempts (7 studies^34,57,68,73,88,104,111^; OR, 1.93; 95% CI, 1.36-2.74; *I*^2^ = 92.0%), as was physical neglect (7 studies^34,57,68,73,88,104,111^; OR 1.79; 95% CI, 1.27-2.53; *I*^2^ = 91.5%), but the overall number of the pooled studies was low. For those studies that did not differentiate between the types of abuse and neglect, we calculated the pooled OR by creating a category termed *overall child abuse*. Children and young adults who had been exposed to any type of abuse and neglect were found to have more than 3-fold increased odds for suicide attempts (10 studies^47,59,60,65,66,75,80,96,99,105^; OR, 3.38; 95% CI, 2.09-5.47; *I*^2^ = 92.6%), but there was an indication of publication bias. The OR corrected for publication bias was 2.91 (95% CI, 2.45-3.44).

**Table 1.** Results of Meta-analyses of the Association Between Forms of Childhood Maltreatment and Suicide Behaviors in Youth

Maltreatment subtype by suicide behavior	No. of studies	No. of participants	Effect size, pooled OR (95% CI)	Heterogeneity		Publication bias	
				*P* value	*I*^2^ value, %	Egger *P* value	Trim and fill, OR (95% CI)
**Suicide attempts**							
Sexual abuse	48	253 638	3.41 (2.90-4.00)	<.001	97.4	.07	NA
Physical abuse	26	125 559	2.18 (1.75-2.71)	<.001	90.2	.01	3.07 (2.95-3.19)
Emotional abuse	6	92 929	2.21 (1.37-3.57)	<.001	95.6	NA	NA
Emotional neglect	7	92 929	1.93 (1.36-2.74)	<.001	92.0	NA	NA
Physical neglect	7	92 929	1.79 (1.27-2.53)	<.001	91.5	NA	NA
Overall child abuse	10	19 882	3.38 (2.09-5.47)	<.001	92.6	.01	2.91 (2.45-3.44)
**Suicidal ideation**							
Sexual abuse	33	188 418	2.46 (2.08-2.90)	<.001	94.6	.12	NA
Physical abuse	23	76 492	1.95 (1.67-2.27)	<.001	81.9	.52	NA
Emotional abuse	7	26 369	1.82 (1.47-2.25)	<.001	88.0	NA	NA
Overall child abuse	7	8225	2.36 (1.98-2.82)	<.001	0.0	NA	NA
**Suicide plans**							
Sexual abuse	7	20 884	4.12 (2.44-6.95)	<.001	77.4	NA	NA

Abbreviations: NA, not applicable; OR, odds ratio.

Sexual abuse was associated with 2.5-fold increased odds for suicide ideation (33 studies^35,36,38,41,42,45,46,48,49,50,51,53,55,61,65,67,69,76,77,78,83,85,92,93,94,95,101,103,104,106,107,109,110^; OR, 2.46; 95% CI, 2.08-2.90; *I*^2^ = 94.6%), and heterogeneity was high. Physical abuse (23 studies^35,45,46,48,51,55,64,67,69,70,71,78,83,85,91,92,93,95,98,103,104,107,109^; OR, 1.95; 95% CI, 1.67-2.27; *I*^2^ = 81.9%), emotional abuse (7 studies^35,45,72,78,79,104,109^; OR, 1.82; 95% CI, 1.47-2.25; *I*^2^ = 88.0%), and overall child abuse (7 studies^39,58,59,65,69,96,99^; OR, 2.36; 95% CI, 1.98-2.82; *I*^2^ = 0.0%) were associated with approximately 2-fold increased odds for suicide ideation, but heterogeneity was high or the number of studies was low in this analysis (funnel plots are shown in eFigure 13 in the Supplement).

Seven individual studies explored the link between sexual abuse and suicide plans in children and young people. The pooled OR indicated that sexual abuse was associated with 4-fold increased odds for suicide plans (7 studies^38,42,44,55,65,76,77^; OR, 4.12; 95% CI, 2.44-6.95; *I*^2^ = 77.4%), and heterogeneity was high.

### Meta-regression Analyses

The number of comparison studies per category allowed us to perform multivariate meta-regression analyses only for the associations between sexual and physical abuse and suicide ideation and suicide attempts (Table 2). We included a number of key covariates, including mean age, percentage of participants identifying as male, type of research design (1 indicates cross-sectional; 2, prospective or experimental), screening tests for childhood maltreatment and suicide behaviors (1 indicates self-report; 2, interview), type of population (1 indicates community; 2, other, which mostly consisted of clinical inpatients or homeless individuals), and quality appraisal score (1 indicates low; 2, high). For the association between sexual abuse and suicide attempts, the results demonstrated that studies that used community samples (β [SE] = −1.68 [0.79]; *P* = .04), were based on younger participants (β [SE] = −0.59 [0.27]; *P* = .03), and had lower methodological quality (β [SE] = −2.86 [1.30]; *P* = .03) reported stronger associations. The overall model was not statistically significant (χ^2^_7_ = 1.56; *P* = .16) but reduced the *I*^2^ value from 97.4% to 49.9%. For the association between sexual abuse and suicide ideation, studies that were based on younger participants (β [SE] = −0.41 [0.18]; *P* = .03) also reported stronger associations. The overall model was not statistically significant (χ^2^_7_ = 1.68; *P* = .16) and reduced the *I*^2^ value from 94.6% to 22.0%. None of the moderators that were examined affected the associations between physical abuse and suicide ideation and suicide attempts.

**Table 2.** Results of the Multivariate Meta-regression Analyses

Maltreatment subtype by suicide behavior	Multivariate regression analyses		
	β (SE)	*P* value	*I*^2^ value, %
**Suicide attempts**			
Sexual abuse			
Mean age	−0.59 (0.27)	.03	49.9
Male, %	−0.02 (0.02)	.23	
Research design (cross-sectional vs prospective)	1.46 (1.21)	.23	
Abuse measure (scale vs interview)	1.23 (1.26)	.24	
Suicide measure (scale vs interview)	1.23 (1.26)	.34	
Population (community vs other)	−1.68 (0.79)	.04	
Critical appraisal score (low vs high)	−2.86 (1.30)	.03	
Physical abuse			
Mean age	−0.29 (0.24)	.24	NA
Male gender (%)	−0.02 (0.02)	.60	
Research design (cross-sectional vs prospective)	0.10 (0.91)	.91	
Abuse measure (scale vs interview)	1.01 (1.67)	.55	
Suicide measure (scale vs interview)	−0.27 (1.57)	.55	
Population (community vs other)	−0.47 (0.41)	.27	
Critical appraisal score (low vs high)	−1.24 (0.90)	.18	
**Suicide ideation**			
Sexual abuse			
Mean age	−0.41 (0.18)	.03	22.0
Male, %	−0.02 (0.02)	.17	
Research design (cross-sectional or prospective)	−0.33 (0.48)	.50	
Abuse measure (scale or interview)	−0.44 (0.55)	.42	
Suicide measure (scale or interview)	−0.04 (0.76)	.96	
Population (community or other)	−0.80 (0.55)	.16	
Critical appraisal score (low or high)	−0.44 (1.19)	.72	
Physical abuse			
Mean age	−0.19 (0.23)	.42	NA
Male, %	−0.01 (0.01)	.61	
Research design (cross-sectional or prospective)	0.29 (0.40)	.47	
Abuse measure (scale or interview)	−0.47 (0.44)	.46	
Suicide measure (scale or interview)	0.52 (0.66)	.45	
Population (community or other)	−0.20 (0.36)	.59	
Critical appraisal score (low or high)	0.48 (0.66)	.59	

Abbreviation: NA, not applicable.

### Leave-One-Out Sensitivity Analyses

The leave-one-out sensitivity analyses did not show any marked differences in the results for the associations between sexual abuse and suicide attempts (OR range, 3.26 [95% CI, 2.82-3.78] to 3.51 [95% CI, 2.95-4.18]), sexual abuse and suicide ideation (OR range, 2.37 [95% CI, 2.01-2.79] to 2.52 [95% CI, 2.13-2.98]), physical abuse and suicide attempts (OR range, 2.00 [95% CI, 1.62-2.45] to 2.21 [95% CI, 1.75-2.79]), physical abuse and suicide ideation (OR range, 1.86 [95% CI, 1.61-2.14] to 1.97 [95% CI, 1.69-2.29]), and overall abuse and suicide attempts (OR range, 3.06 [95% CI, 1.90-4.92] to 3.68 [95% CI, 2.16-6.29]). These results lent confidence for the robustness of the findings.

## Discussion

This is the first comprehensive meta-analytic review, to our knowledge, to explore the association between core types of childhood maltreatment and suicide experiences in children and young people. With 57 more studies than the most recent review,^16^ the present meta-analysis combined data from 79 studies based on 337 185 participants. A key, and novel, contribution of this review is that it establishes the experiences of the core forms of childhood maltreatment (ie, sexual, physical, and emotional abuse and physical and emotional neglect) as critical lifetime events that are associated with increased odds for suicide attempts in children and young adults to 24 years of age. The pooled ORs were positive and significant for all the comparisons examined and ranged from 1.79 to 3.41 for suicide attempts. The present study differs from previous meta-analyses^16^ in that it (1) incorporated a larger pool of studies to allow for examination of the associations for a broader scope of childhood maltreatment, rather than focusing exclusively on childhood sexual and physical abuse; (2) was the first, to our knowledge, to examine and confirm that studies with lower methodological quality did not necessarily influence the strength of these associations; and (3) was the first, to our knowledge, to examine the association between core forms of childhood maltreatment and suicide ideation and plans.

Our findings demonstrated that the experiences of childhood sexual, physical, and emotional abuse were associated with as much as 2.5-fold greater odds for suicide ideation and that sexual abuse was associated with 4.0-fold increased odds for suicide plans in young people. We were unable to find research that directly linked suicide plans with the other core forms of childhood abuse and/or neglect. Overall, these are important findings because suicide plans, especially when they occur during peak suicide ideation, can lead to suicide attempts and deaths by suicide.^112^ Clearly, more research examining the links between suicide ideation, plans, and core types of childhood maltreatment needs to be undertaken.

A prime aim of this review was to explore the influence of key sample and study characteristics on the strength of the association between core types of childhood abuse and suicide behavior in children and young people. There were 3 primary findings. First, the association between suicide attempts and childhood sexual abuse was stronger for younger people from the community with unknown mental and/or physical health problems compared with those who had received a formal diagnosis or treatment for mental health problems or had experienced additional life stressors (eg, homelessness, running away from home) and for studies with a lower methodological quality. Second, young age was also associated with a substantially higher likelihood for suicide ideation in people who were sexually abused during childhood. Finally, none of the other examined moderators were found to affect the associations between sexual and physical abuse and suicide ideation and attempts.

Overall, these results provide compelling evidence of the association between core types of childhood maltreatment and suicide experiences in children and young people. Our findings are consistent with those published in previous systematic reviews or meta-analyses.^16,17,18,19,20^ However, this study has advanced this literature by making 3 unique contributions. First, with a total number of 79 studies, this is the most comprehensive meta-analytic review to quantify and report the ORs for the association between core types of childhood maltreatment and suicide attempts. Second, this review is the first, to our knowledge, to provide quantifiable evidence of the strong associations between experiences of childhood maltreatment and suicide ideation and between childhood sexual abuse and suicide plans. A third important contribution is the identification of key sample variables that moderated the associations between childhood sexual maltreatment and suicide attempts and ideation in young individuals. In particular, we demonstrate that childhood sexual abuse was associated more strongly with suicide attempts in young children who were not under the care of clinicians. This finding has important clinical implications in that it highlights an urgent need for incorporating suicide prevention strategies into treatment planning for those young children who have experienced abuse. Furthermore, we found that a stronger association between childhood sexual abuse and suicide ideation also exists in younger individuals. One explanation for this finding is that earlier experiences of sexual abuse may be associated with greater repetition or greater severity of abuse.^113^ Another plausible interpretation is that older and more experienced individuals may be more resilient in dealing with life stressors.^114^ In accord with the latter explanation, research suggests that poorer problem-solving abilities are highly associated with suicide attempts^115^ and that those who attempt suicide tend to perceive themselves as passive problem solvers.^116^ Clearly, more research needs to be conducted in examining which of these explanations are more viable. These findings could be beneficial to clinicians charged with providing treatment aimed at ameliorating the effects of childhood maltreatment for younger children.

### Limitations

There were 3 key limitations of the analysis that warrant discussion. First, heterogeneity was high across most of the comparison groups. We therefore applied random-effects models and performed multivariate meta-regression analyses whenever possible. Although we identified important sources of variation that substantially reduce the heterogeneity contributing to the associations between childhood sexual abuse and suicide attempts and ideation, the modest number of comparison studies across the remaining childhood maltreatment subtypes prevents us from exploring additional sources of variance by running meta-regression analyses. Second, an indication of publication bias was found for the associations between physical and overall child abuse and suicide attempts. We used the trim and fill approach, which substantially increased the effect size for the association between physical abuse and suicide attempts, suggesting that publication bias might not threaten the validity of these results.^117^ The effect size was decreased for the association between overall child abuse and suicide attempts. These results suggest that these findings should be interpreted with caution. Third, in this review, we focused exclusively on core childhood maltreatment types, namely sexual, physical, and emotional abuse and/or neglect, because they have been suggested to play a key role in suicide ideation and attempts.^3,118^ However, additional meta-analyses that use broader criteria to incorporate a larger pool of studies exploring such adverse experiences as violence, bullying, parental deaths, and divorces are clearly needed. Although not a limitation of the present review, we highlight the fact that because most of the included studies had used a cross-sectional research design, our analysis does not imply causality. Studies that use prospective designs that can identify the temporal succession of exposure to the various maltreatment subtypes and the subsequent development of suicide behaviors and/or diary studies that focus on the perceptions and/or memories between abuse and/or neglect and suicide behaviors are crucial to advancing our knowledge in this area.

## Conclusions

With a total number of 79 studies performed from 1989 to 2019, this is, to our knowledge, the most comprehensive systematic review and meta-analysis to date exploring the association between core types of childhood maltreatment and suicide behaviors. The review confirmed evidence of this important association in children and young adults to 24 years of age. Overall, these data suggest that childhood maltreatment is a central social welfare problem that may lead to suicide behaviors. Therefore, research, clinical, and policy actions should be taken with a particular focus on (1) raising public awareness, (2) informing existing policies, and (3) amending treatment protocols for achieving optimal results with respect to childhood maltreatment.

## Notes

Supplement. **eFigure 1.** Forest Plot of the Association Between Sexual Abuse and Suicide Attempts **eFigure 2.** Forest Plot of the Association Between Physical Abuse and Suicide Attempts **eFigure 3.** Forest Plot of the Association Between Emotional Abuse and Suicide Attempts **eFigure 4.** Forest Plot of the Association Between Emotional Neglect and Suicide Attempts **eFigure 5.** Forest Plot of the Association Between Physical Neglect and Suicide Attempts **eFigure 6.** Forest Plot of the Association Between Combined Abuse and Suicide Attempts **eFigure 7.** Forest Plot of the Association Between Sexual Abuse and Suicidal Ideation **eFigure 8.** Forest Plot of the Association Between Physical Abuse and Suicidal Ideation **eFigure 9.** Forest Plot of the Association Between Emotional Abuse and Suicidal Ideation **eFigure 10.** Forest Plot of the Association Between Combined Abuse and Suicidal Ideation **eFigure 11.** Forest Plot of the Association Between Sexual Abuse and Suicidal Plans **eFigure 12.** Funnel Plot of the Association Between Childhood Maltreatment and Suicide Attempts **eFigure 13.** Funnel Plot of the Association Between Childhood Maltreatment and Suicidal Ideation **eTable.** Descriptive Characteristics of the Included Studies **eReferences.**
