# Supplementary material for: Association of Childhood Maltreatment With Suicide Behaviors Among Young People: A Systematic Review and Meta-analysis
Source: JAMA Netw Open. 2020 Aug 5;3(8):e2012563. doi: 10.1001/jamanetworkopen.2020.12563 (PMC7407092; doi:10.1001/jamanetworkopen.2020.12563)
Supplement: Supplement. — eFigure 1. Forest Plot of the Association Between Sexual Abuse and Suicide Attempts eFigure 2. Forest Plot of the Association Between Physical Abuse and Suicide Attempts eFigure 3. Forest Plot of the Association Between Emotional Abuse and Suicide Attempts eFigure 4. Forest Plot of the Association Between Emotional Neglect and Suicide Attempts eFigure 5. Forest Plot of the Association Between Physical Neglect and Suicide Attempts eFigure 6. Forest Plot of the Association Between Combined Abuse and Suicide Attempts eFigure 7. Forest Plot of the Association Between Sexual Abuse and Suicidal Ideation eFigure 8. Forest Plot of the Association Between Physical Abuse and Suicidal Ideation eFigure 9. Forest Plot of the Association Between Emotional Abuse and Suicidal Ideation eFigure 10. Forest Plot of the Association Between Combined Abuse and Suicidal Ideation eFigure 11. Forest Plot of the Association Between Sexual Abuse and Suicidal Plans eFigure 12. Funnel Plot of the Association Between Childhood Maltreatment and Suicide Attempts eFigure 13. Funnel Plot of the Association Between Childhood Maltreatment and Suicidal Ideation eTable. Descriptive Characteristics of the Included Studies eReferences. [file jamanetwopen-3-e2012563-s001.pdf]

Supplementary Online Content

Angelakis I, Austin JL, Gooding P. Association of childhood maltreatment with suicide behaviors among young people: a systematic review and meta-analysis. *JAMA Netw Open*. 2020;3(8):e2012563. doi:10.1001/jamanetworkopen.2020.12563

eFigure 1. Forest Plot of the Association Between Sexual Abuse and Suicide Attempts
eFigure 2. Forest Plot of the Association Between Physical Abuse and Suicide Attempts
eFigure 3. Forest Plot of the Association Between Emotional Abuse and Suicide Attempts
eFigure 4. Forest Plot of the Association Between Emotional Neglect and Suicide Attempts
eFigure 5. Forest Plot of the Association Between Physical Neglect and Suicide Attempts
eFigure 6. Forest Plot of the Association Between Combined Abuse and Suicide Attempts
eFigure 7. Forest Plot of the Association Between Sexual Abuse and Suicidal Ideation
eFigure 8. Forest Plot of the Association Between Physical Abuse and Suicidal Ideation
eFigure 9. Forest Plot of the Association Between Emotional Abuse and Suicidal Ideation
eFigure 10. Forest Plot of the Association Between Combined Abuse and Suicidal Ideation
eFigure 11. Forest Plot of the Association Between Sexual Abuse and Suicide Plans
eFigure 12. Funnel Plot of the Association Between Childhood Maltreatment and Suicide Attempts
eFigure 13. Funnel Plot of the Association Between Childhood Maltreatment and Suicidal Ideation
eTable. Descriptive Characteristics of the Included Studies
eReferences.

This supplementary material has been provided by the authors to give readers additional information about their work.

eFigure 1. Forest Plot of the Association Between Sexual Abuse and Suicide Attempts

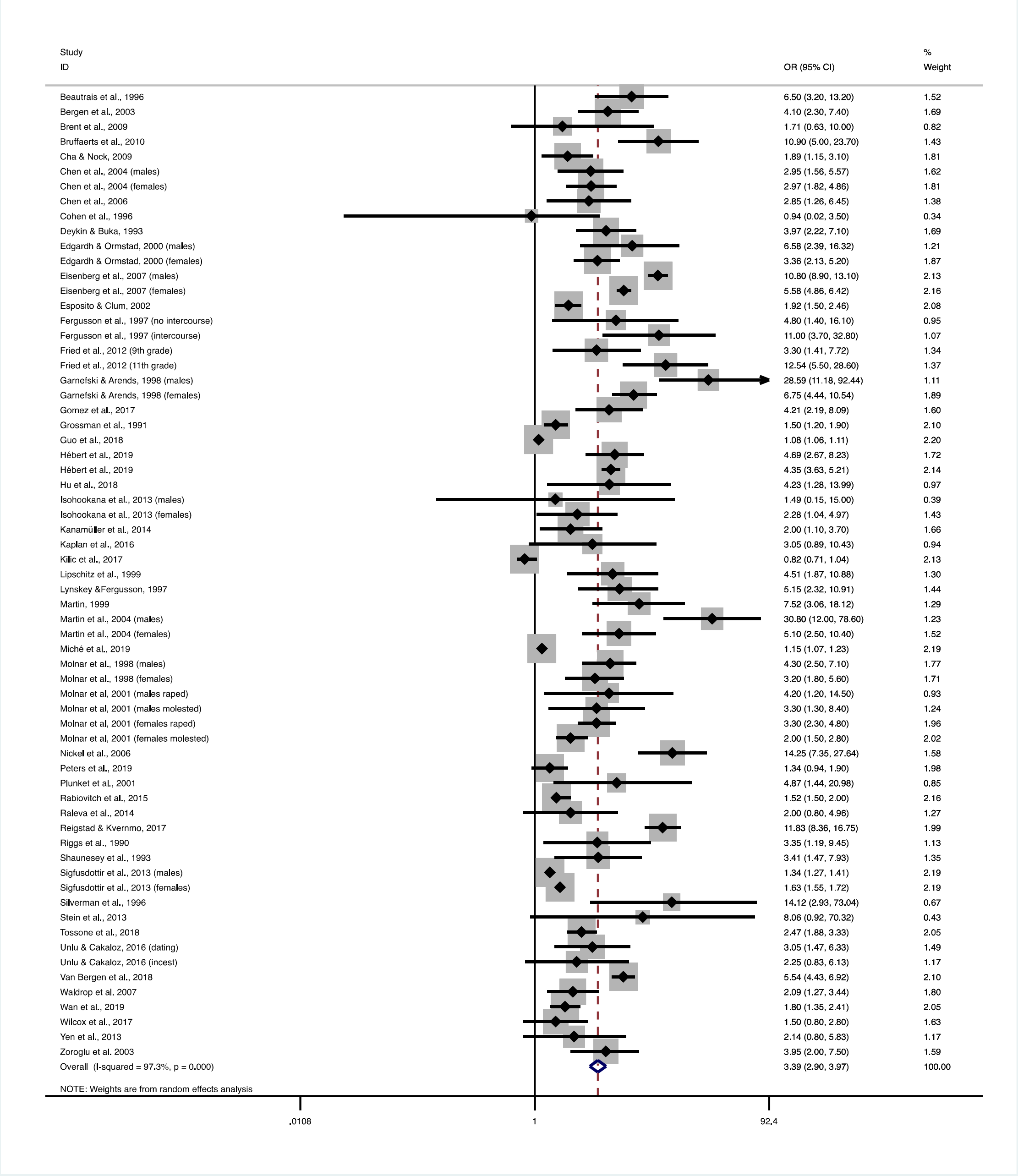

Note random effects model used. OR = Odds ratio.

©2020 Angelakis I et al. *JAMA Network Open*.

eFigure 2. Forest Plot of the Association Between Physical Abuse and Suicide Attempts

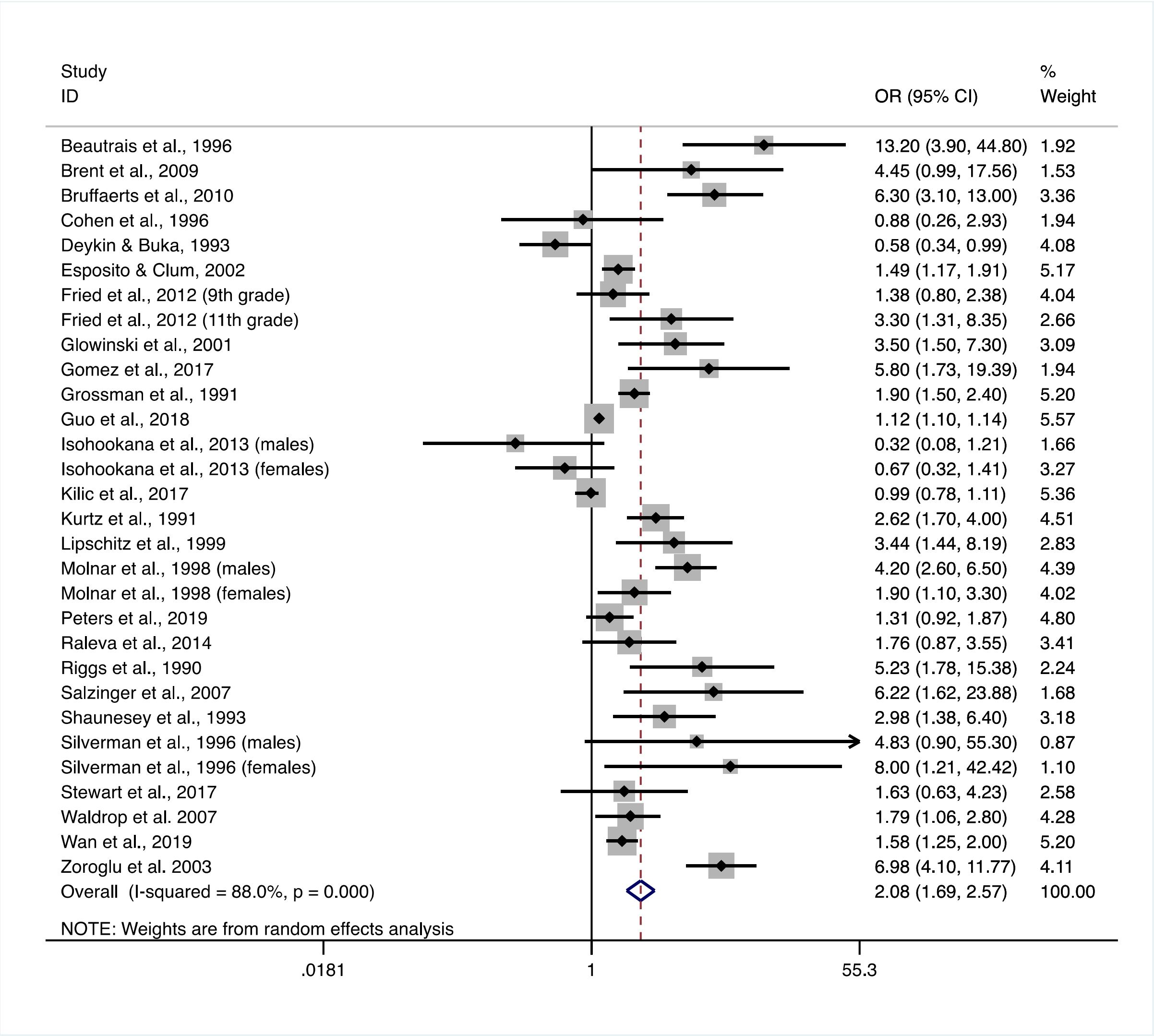

Note random effects model used. OR = Odds ratio.

©2020 Angelakis I et al. *JAMA Network Open*.

eFigure 3. Forest Plot of the Association Between Emotional Abuse and Suicide Attempts

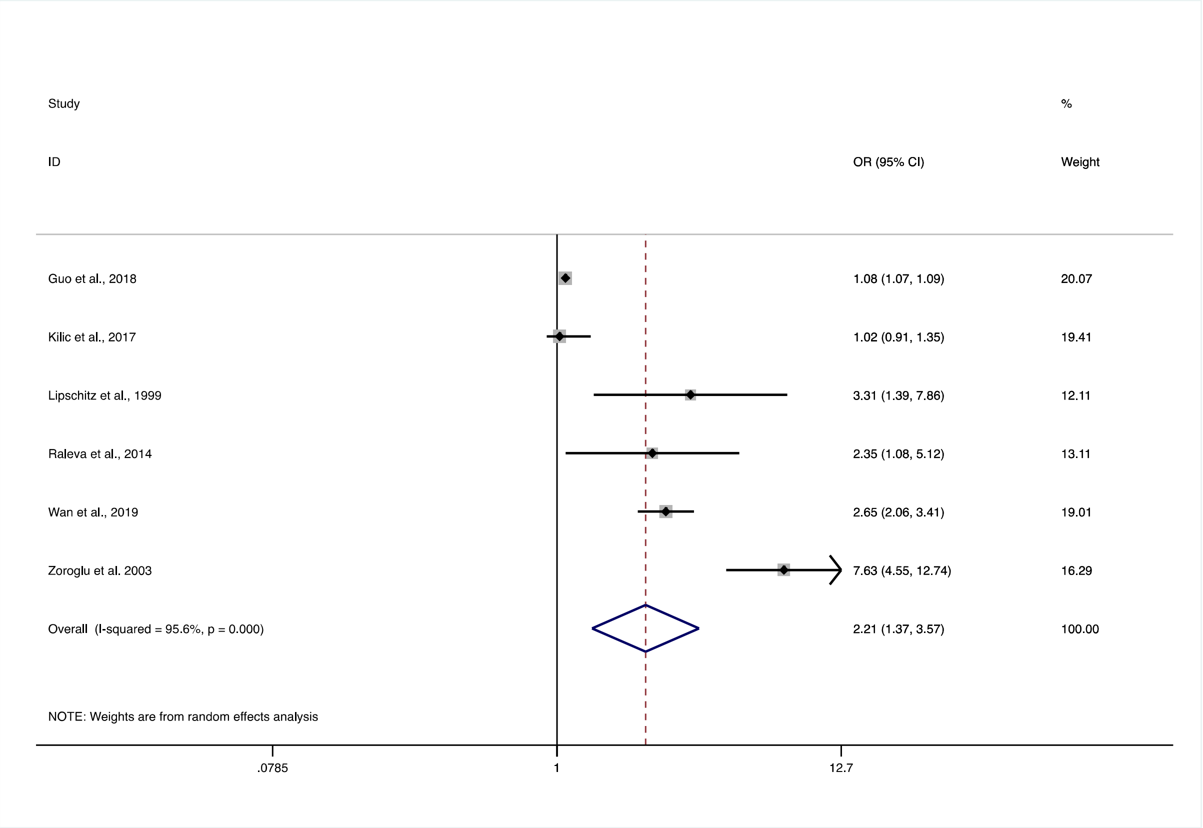

Note random effects model used. OR = Odds ratio.

©2020 Angelakis I et al. *JAMA Network Open*.

eFigure 4. Forest Plot of the Association Between Emotional Neglect and Suicide Attempts

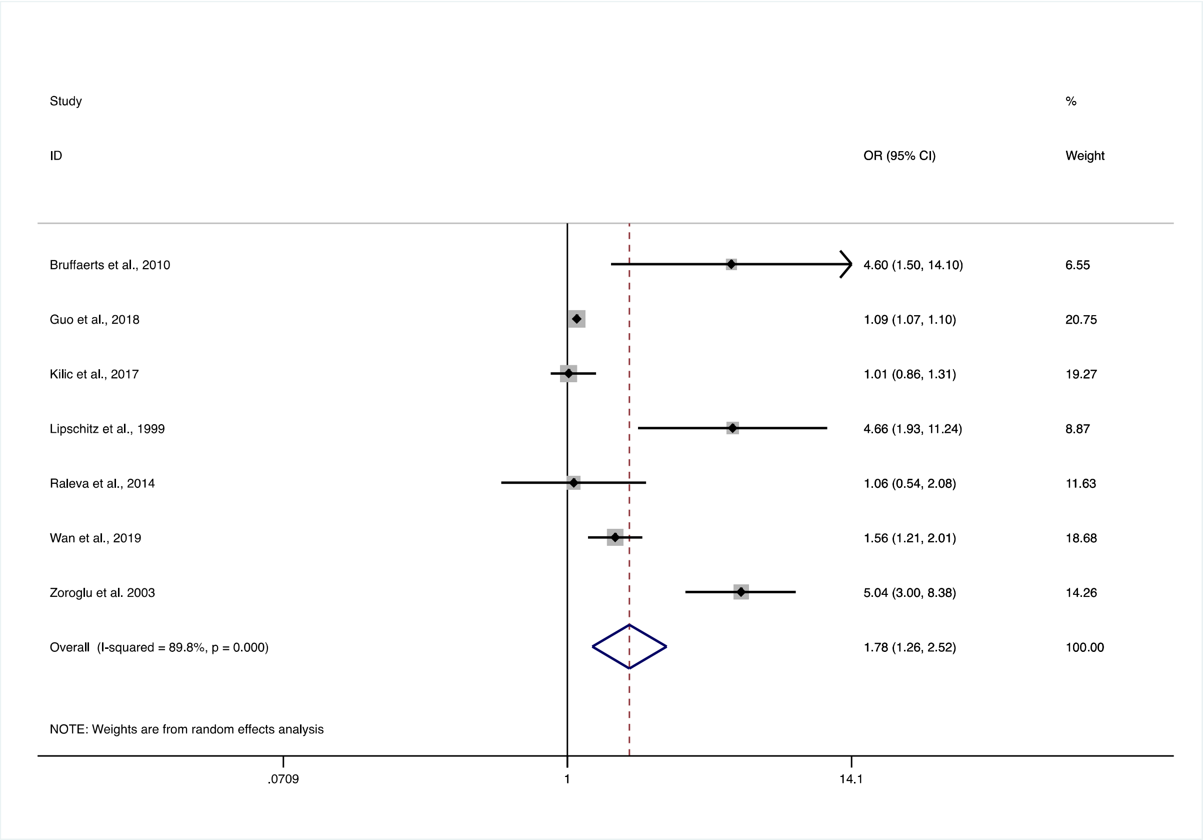

Note random effects model used. OR = Odds ratio.

©2020 Angelakis I et al. *JAMA Network Open*.

eFigure 5. Forest Plot of the Association Between Physical Neglect and Suicide Attempts

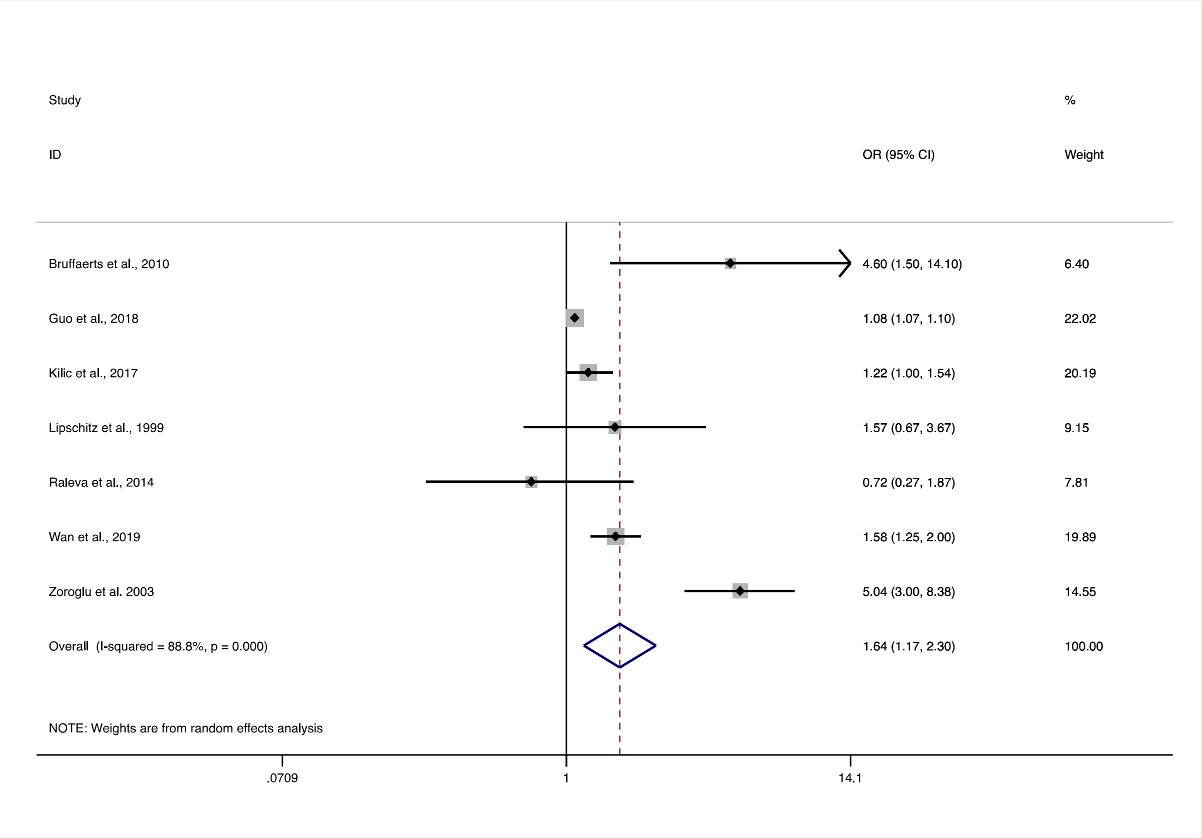

Note random effects model used. OR = Odds ratio.

©2020 Angelakis I et al. *JAMA Network Open*.

eFigure 6. Forest Plot of the Association Between Combined Abuse and Suicide Attempts

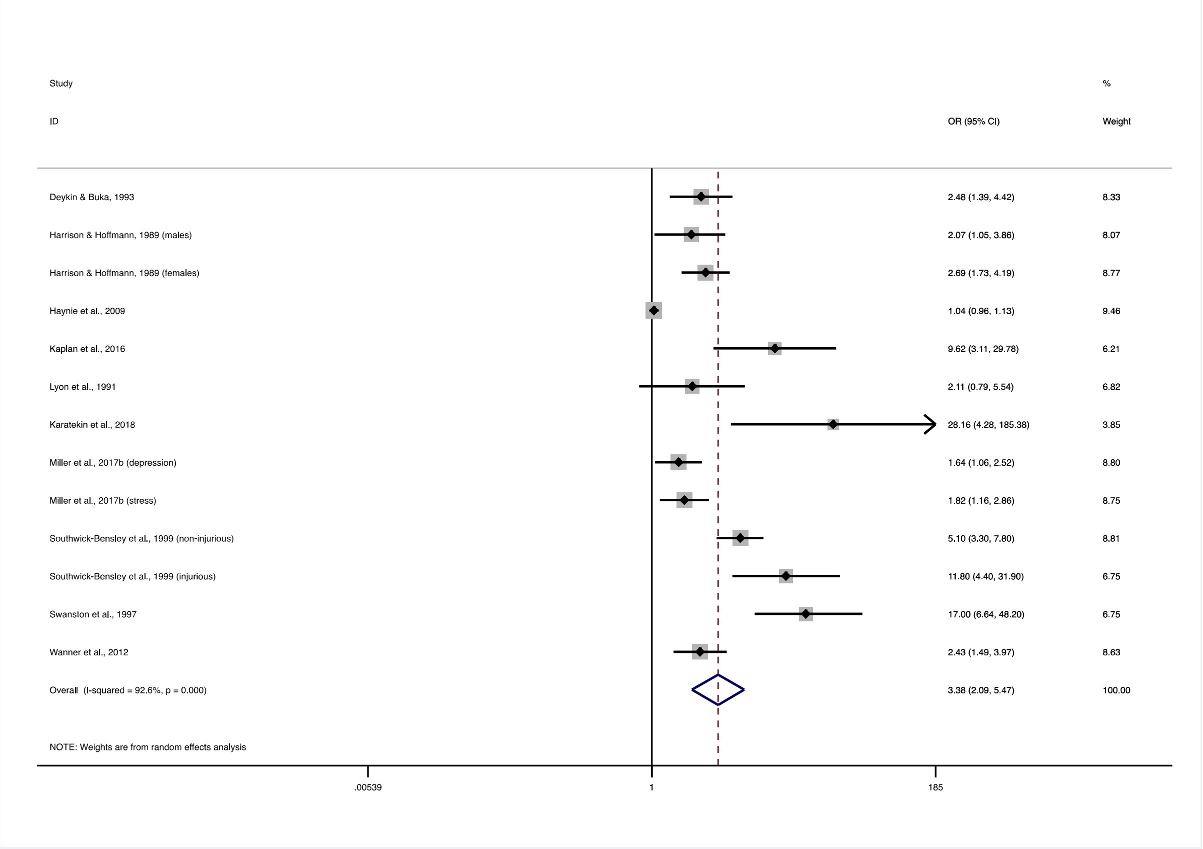

. Note random effects model used. OR = Odds ratio.

eFigure 7. Forest Plot of the Association Between Sexual Abuse and Suicidal Ideation

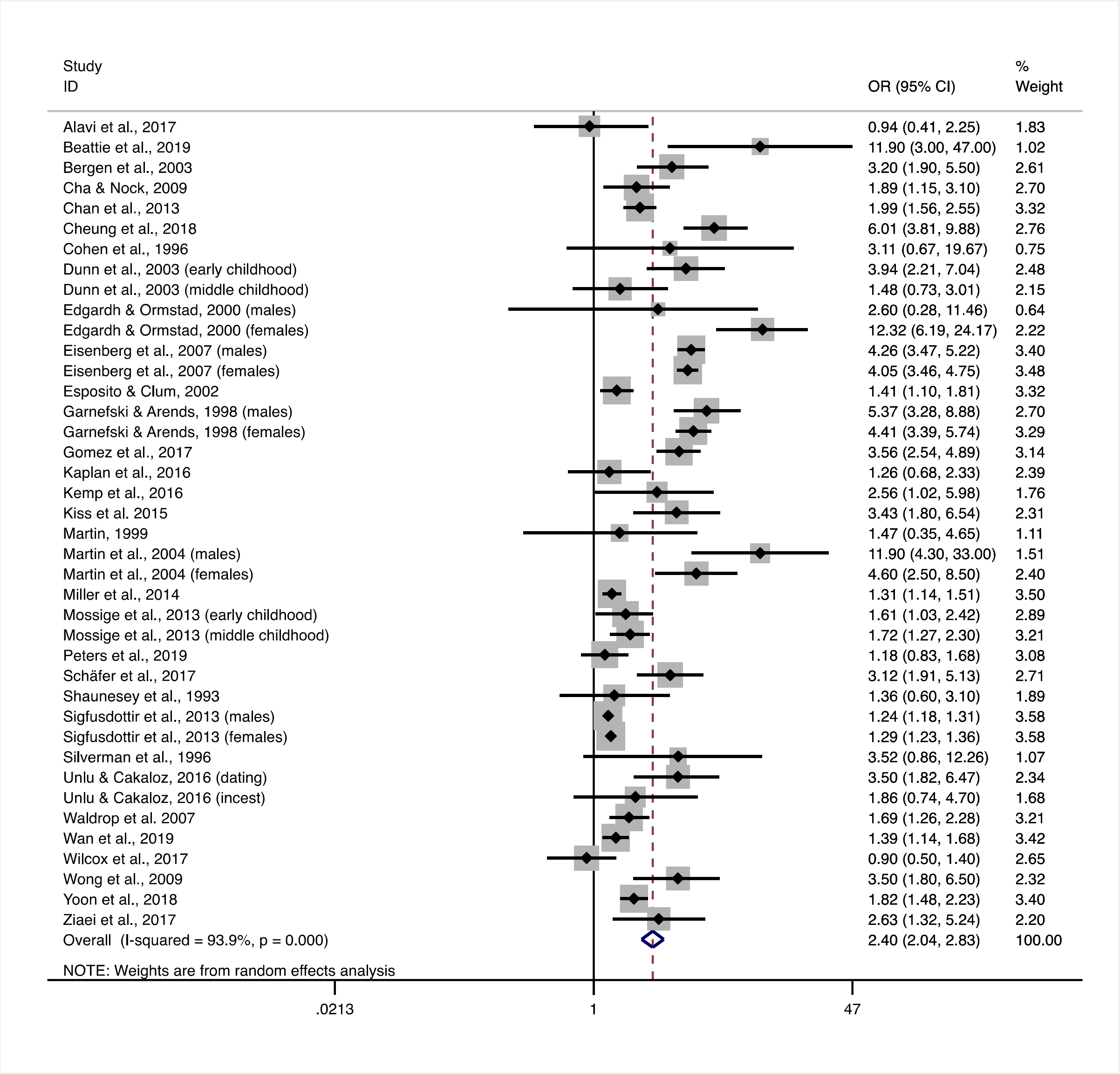

Note random effects model used. OR = Odds ratio.

©2020 Angelakis I et al. *JAMA Network Open*.

eFigure 8. Forest Plot of the Association Between Physical Abuse and Suicidal Ideation

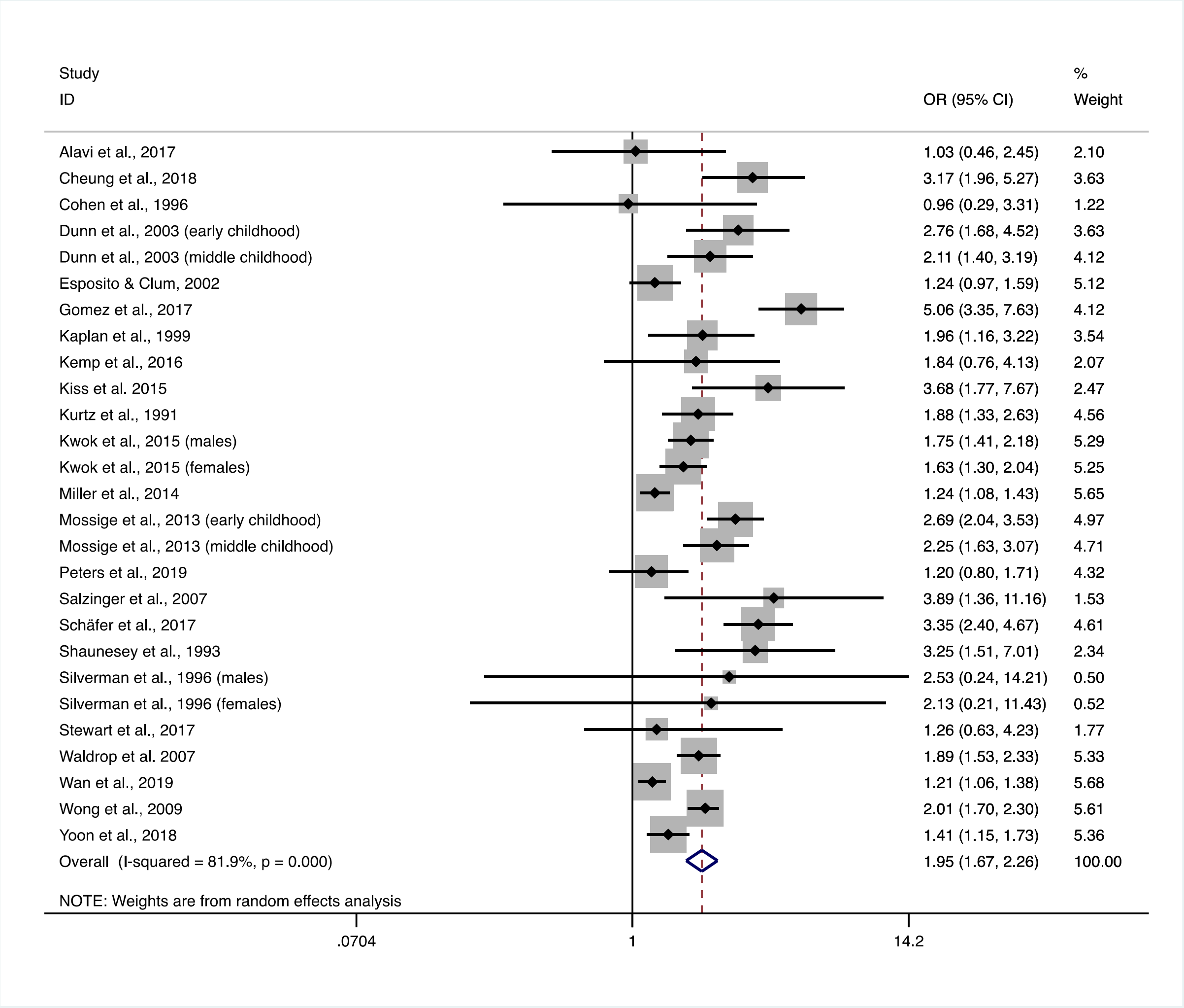

Note random effects model used. OR = Odds ratio.

©2020 Angelakis I et al. *JAMA Network Open*.

eFigure 9. Forest Plot of the Association Between Emotional Abuse and Suicidal Ideation

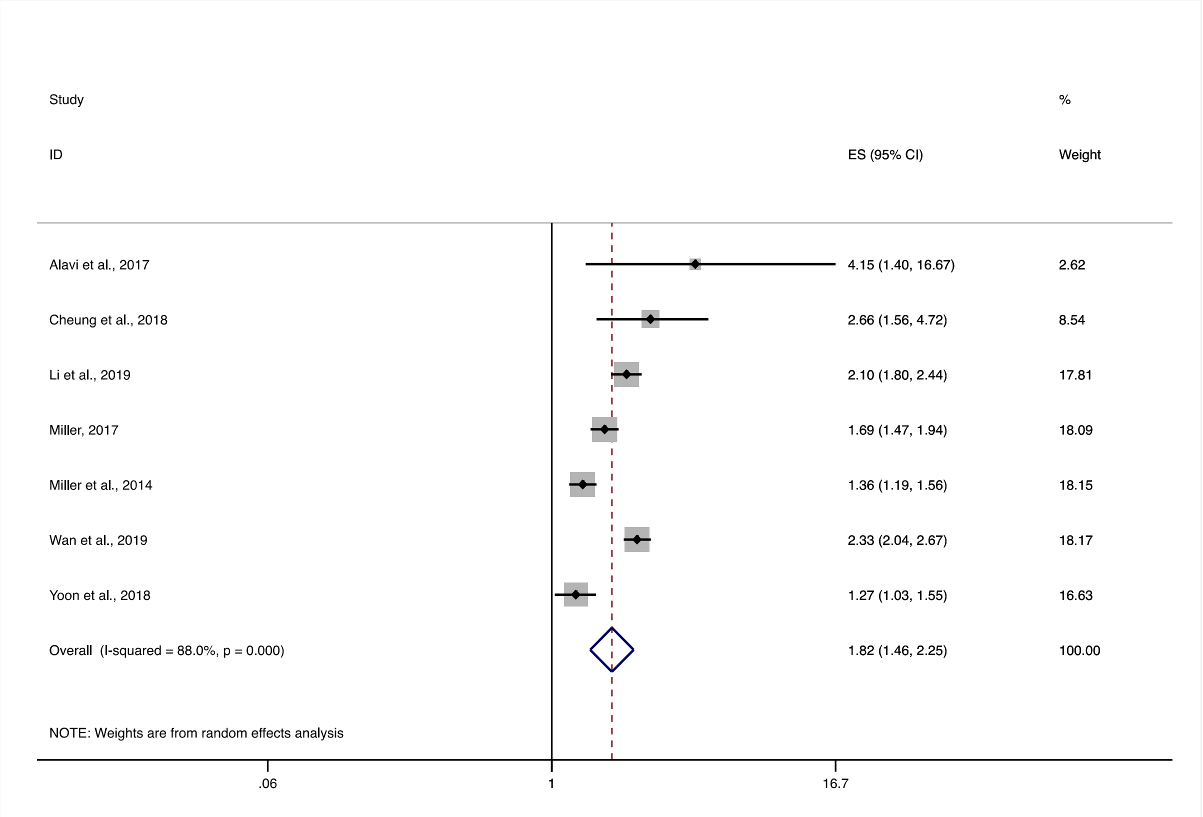

Note random effects model used. OR = Odds ratio.

©2020 Angelakis I et al. *JAMA Network Open*.

eFigure 10. Forest Plot of the Association Between Combined Abuse and Suicidal Ideation

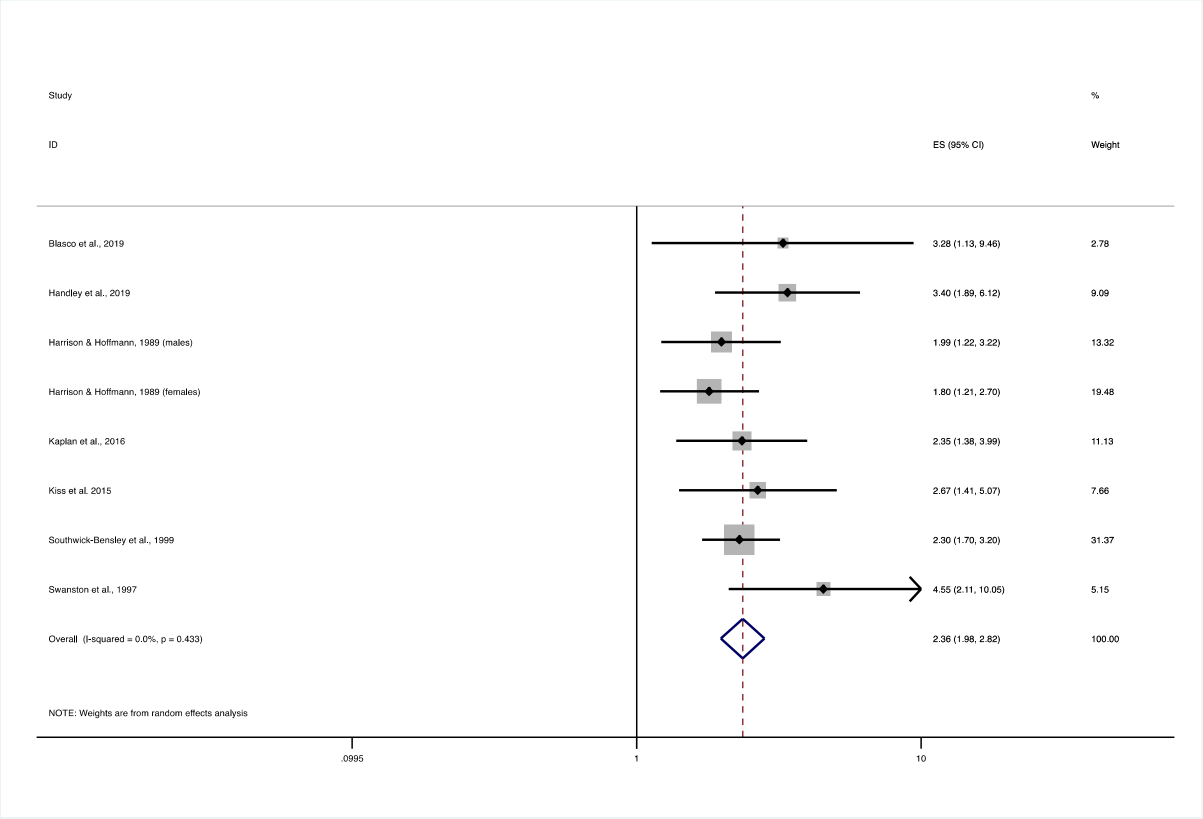

Note random effects model used. OR = Odds ratio.

©2020 Angelakis I et al. *JAMA Network Open*.

eFigure 11. Forest Plot of the Association Between Sexual Abuse and Suicide Plans

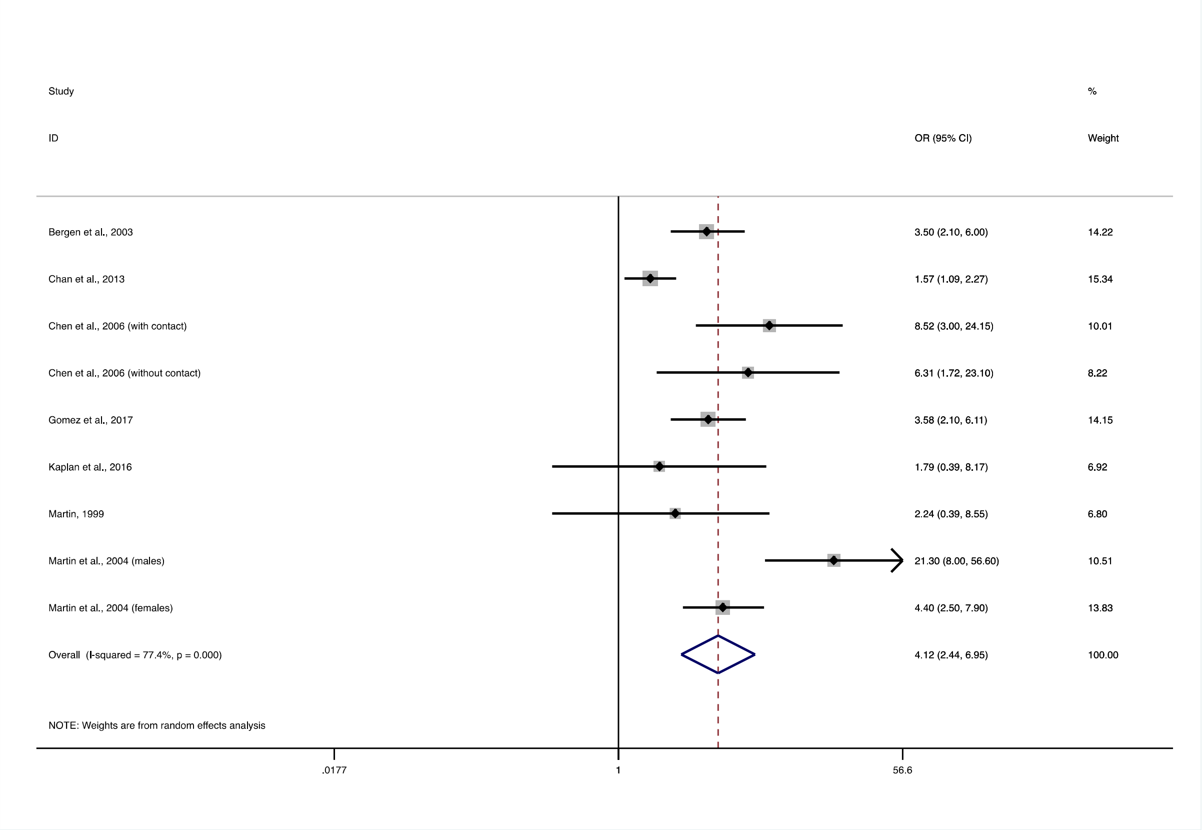

Note random effects model used. OR = Odds ratio.

©2020 Angelakis I et al. *JAMA Network Open*.

eFigure 12. Funnel Plots of the Association Between Childhood Maltreatment and Suicide Attempts

A

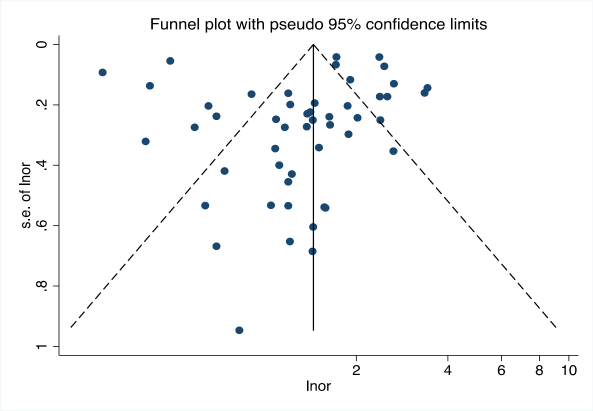

B

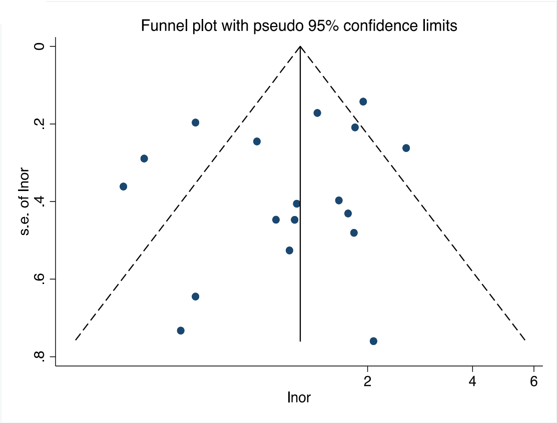

C

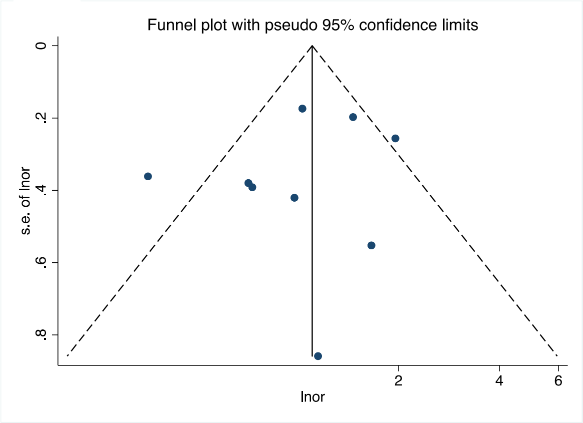

: (A) sexual abuse, (B) physical abuse, (C) Overall abuse

©2020 Angelakis I et al. *JAMA Network Open*.

eFigure 13. Funnel Plots of the Association Between Childhood Maltreatment and Suicidal Ideation

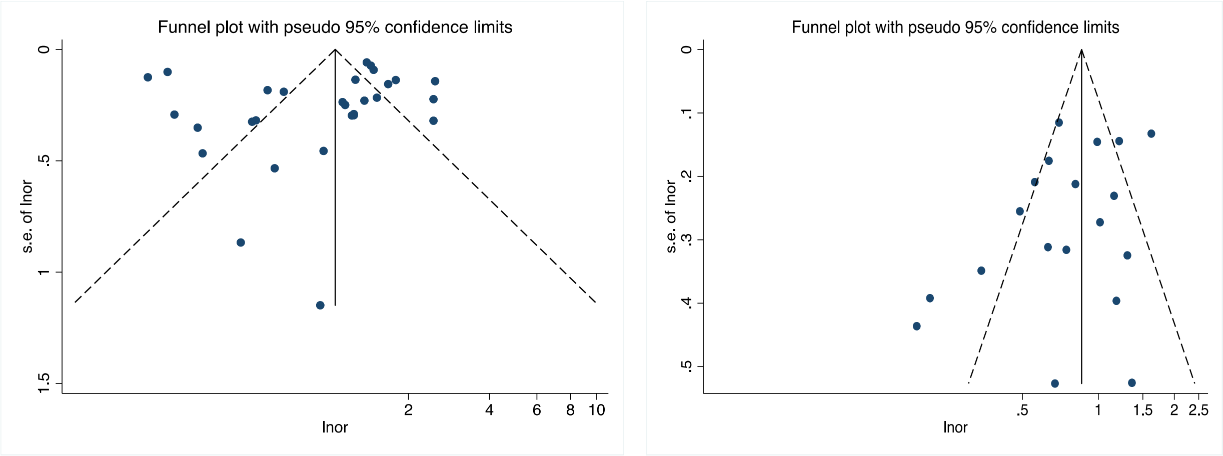

(A) sexual abuse, and (B) physical maltreatment.

©2020 Angelakis I et al. *JAMA Network Open*.

eTable. Descriptive Characteristics of the Included Studies

Study	**Country**	**Study Design**	**Screening tool for abuse**	**Mode of childhood abuse**	**Screening tool for suicidality**	**Mode of suicidality**	**Timeframe**	**Sample Size *N***	**Men (%)**	**Age (years)**	**Population**	**Q.A.**
Alavi et al., 2017[1]	Canada	R	MC	Emotional, physical & sexual abuse	MC	SI	Lifetime	270, response rate = 100%	38.51%	M_{age} = 14.4, range: 8-17	Cli.S.	3/5
Beattie et al., 2019[2]	India	CS	SRQ	Sexual abuse	SRQ	SI	Current	1,191, response rate = 100%	0%	Range: 13-14	Com.S.	2/5
Beautrais et al., 1996[3]	New Zealand	CS	CI	Sexual & physical abuse	SRQ	SA	n/r	132, response rate = 97.7%	45.7%	M_{age} = 19.4, range: 13-24	MS	3/5
Bergen et al., 2003[4]	Australia	CS	SRQ	Sexual abuse	SRQ	SI, SP, ST & SA	Lifetime	2,603, response rate = 85%	55.4%	M_{age} = 13	Com.S.	2/5
Blasco et al., 2019[5]	Spain	FU	ASEQ	Sexual & physical abuse	SRQ	SI	Current	T2: 1,248, response rate = 53.82%	44%	Range: 18-24	Com.S	2/5
Brent et al., 2009[6]	United States	FU	CEQ	Sexual & physical abuse	SSRS	SA	Current	T2: 119, response rate: 96%	22.6%	M_{age} = 15.8, range: 12-18	Cli.S.	3/6
Bruffaerts et al., 2010[7]	Surveys were carried out in 21 countries	CS	CI	Sexual, physical abuse, & neglect	CIDI	SI, SP & SA	Lifetime	109,377, response rate = 73.3%	n/r	n/r	Cli.S.	3/5
Cha & Nock, 2009[8]	United States	CS	CTQ	All forms of abuse/neglect	SITBI	SI & SA	Past year	94, response rate = 57.45%	14.8%	M_{age} = 17.30, range: 12-19	MS	2/5
Chan et al., 2013[9]	Malaysia	CS	SRQ	Sexual abuse	SRQ	SI & SP	Lifetime	6,786, response rate = 61.5%	47.5%	M_{age} = 17.68, range: 17-18	CYC	1/5
Chen et al., 2004[10]	China	CS	SRC	Sexual abuse	YRBS	SI & SP	Past year	3,262, response rate = 70.5%	49.8%	M_{age} = 17.2, range:16-24	Com.S	3/5
Chen et al., 2006[11]	China	R	SRC	Sexual abuse	YRBS	SI & SP	Past year	435, response rate = 80.7%	0%	M_{age} = 17.6, range: 16-23	Com.S.	2/5
Cheung et al., 2018[12]	United States	CS	NCS-A as adapted by the CTS	All forms of abuse & physical neglect	CI	SI	Past year	10,123, response rate: 80.7%	n/r	Range: 13-17	Com.S.	3/5

©2020 Angelakis I et al. *JAMA Network Open*.

Cohen et al., 1996[13]	United States	CS	SRQ	Sexual & physical abuse	n/r	SI, ST & SA	n/r	105, response rate: 100%	30.48%	M_{age} = 14.7, range: 12-18	Cli.S.	1/5
Deykin & Buka, 1993[14]	United States	CS	DIS	Sexual & physical abuse	DIS	SI & SA	Lifetime	300, response rate: 100%	91.33%	Range: 15-19	Cli.S.	3/5
Dunn et al., 2013[15]	United States	FU	SRQ	Sexual & physical abuse	SRQ	SI	Past year	T2: 20,745, response rate: 69%	50.85%	M_{age} = 15.95,	Com.S.	4/6
Edgardh & Ormstad, 2000[16]	Sweden	CS	SRQ	Sexual abuse	SRQ	SI & SA	Lifetime	1,943, response rate = 92.2%	41.9%	17 years old	Com.S.	1/5
Eisenberg et al., 2007[17]	United States	CS	MSS	Sexual abuse	SRQ	SI & SA	Lifetime	131,862, response rate = 63.5%	49.1%	M_{age} = 14	Com.S.	1/5
Esposito & Clum, 2002[18]	United States	CS	CAS & CMS	Sexual & physical abuse	SSB	SI & SA	Lifetime & current	213, response rate = 93.9%	70.5%	M_{age} = 15.7, range: 12-17	Incar.Ind.	2/5
Fried et al., 2012[19]	United States	FU	SRQ	Sexual & physical abuse	SRQ	SA	Past year	T2: 27,000, response rate = 12.5%	9th: 48.6% 11th: 50.4%	M_{age} = 15.84	Com.S.	2/6
Garnefski & Arends, 1998[20]	Netherlands	CS	SRQ	Sexual abuse	SRQ	SI & SA	Lifetime & current	12,599, response rate = 11.83%	25.24%	M_{age} = 15.00, range: 12-19	Com.S.	0/5
Glowinski et al., 2000[21]	United States	CS	An adaption of the C-SSAGA interview	Physical abuse	CI	SA	Lifetime	3,416, response rate = 85%	0%	M_{age} = 15.5	Com.S.	4/5
Gomez et al., 2017[22]	United States	CS	CIDI	Sexual & physical abuse	CIDI	SI, SP & SA	Lifetime	10,148, response rate = 91.37%	51.41%	Range: 13-18	Com.S.	4/5
Grossman et al., 1991[23]	United States	CS	NAHS	Sexual & physical abuse	NAHS	SA	Lifetime	7,241, response rate = 92%	49%	M_{age} = 14.4	Com.S.	2/5
Guo et al., 2018[24]	China	CS	CTQ	All forms of abuse/neglect	SRQ	SA	Past year	83,203, response rate = 91%	44.8%	M_{age} = 16.6	Com.S.	2/5
Handley et al., 2019[25]	USA	CS	CTQ	All forms of abuse/neglect	KSADS	SI	Current	164, response rate = 100%	0%	M_{age} = 14	Cli.S.	
Harrison & Hoffman, 1989[26]	United States	CS	CI	Sexual & physical abuse	CI	SI & SA	Current	1,824, response rate = 77.58	68.62%	M_{age} = 15.9	Cli.S.	3/5
Haynie et al., 2009[27]	United States	FU	SRQ	Sexual & physical abuse	CI	SA	Past year	T2:11,997, response rate = 99.6%	39.5% - 55.7%	M_{age} = 15.23	Com.S.	4/6

©2020 Angelakis I et al. *JAMA Network Open*.

Hébert et al., 2019[28]	Canada	CS	SRQ	Sexual abuse	SRQ	SI & SA	Lifetime	8,230, response rate: 99.56%	42.2%	M_{age} = 15.35	Com.S.	2/5
Hu et al., 2018[29]	Taiwan	CS	MC	Sexual abuse	MC	SA	Current	110, response rate = 82.73%	3.3%	M_{age} = 13.31	Cli.S.	2/5
Isohookana et al., 2013[30]	Finland	FU	K-SADS-PL	Sexual & physical abuse	K-SADS-PL	SA	Current	T2: 637, response rate = 79.7%	40.9%	M_{age} = 15.4, range: 12-17	Cli.S.	4/6
Kaplan et al., 1999[31]	United States	CS	SR	Physical abuse	SPS	SI	Current	199, response rate = 100%	50%	Median: 15-16	MS	2/5
Kaplan et al., 2016[32]	United States	FU	CTQ	All forms of abuse/neglect	SITBI	SI, SP & SA	Lifetime & current	T1: 58, response rate = 100% T2: 40, response rate = 69%	0%	M_{age} = 17.2	MS	4/6
Karatekin et al., 2018[33]	United States	FU	MCR	Combined childhood maltreatment	CCS	SA	Lifetime	812, response rate = 64%	45%	M_{age} = 7.7	MS	4/6
Kemp et al., 2016[34]	United States	R	FI	Sexual & physical abuse	FI	SI & SA	Lifetime	454, response rate = 77.8%	61%	M_{age} = 15, range: 10-18	JO	3/5
Kilic et al., 2017[35]	Turkey	CS	CTQ	All forms of abuse/neglect	SRQ	SA	Lifetime	207, response rate = unclear	44.5%	M_{age} = 15, range: 11-18	MS	2/5
Kiss et al. 2015[36]	Cambodia, Thailand and Vietnam	CS	CI	Sexual & physical abuse	CI	SI & SA	Current	395, response rate = 98%	18%	M_{age} = 15, range: 10-17	VT	4/5
Kurtz et al., 1991[37]	United States	CS	CIR	Sexual & physical abuse	CIR	SI & SA	Current	2,019, response rate = n/r	50%	M_{age} = 14.5	RY	2/5
Kwok et al., 2015[38]	China	CS	CTS-PC	Physical abuse	C-SIS	SI	Lifetime	566, response rate = 93%	46.6%	M_{age} = 14, range: 12-17	Com.S.	1/5
Li et al., 2019[39]	China	FU	PRCAS	Emotional abuse	SBQ-R	SI	Current	3,555, response rate = 63.54	46.2%	M_{age} = 15.11	Com.S.	
Lipschitz et al., 1999[40]	United States	CS	CTQ	All forms of abuse/neglect	TEQ-A	SA	Lifetime	71, response rate = 100%	47.8%	M_{age} = 14.8, range: 12-18	Cli.S.	1/5
Lynskey &Ferusson, 1997[41]	New Zealand	FU	CI	Sexual abuse	CIDI	SA	Current	T2:1,265, response rate = 81%	n/r	n/r	Com.S.	4/6
Lyon et al., 1991[42]	United States	CS	PCC	Abuse	SRC/MC	SA	Current	175, response rate = 65.14%	n/r	M_{age} = 14.9, range: 12-17	MS	3/5

©2020 Angelakis I et al. *JAMA Network Open*.

Martin, 1996[43]	Australia	CS	SRQ	Sexual abuse	SRQ	SI, SP & SA	Current	419, response rate = 84%	71%	M_{age} = 15.2, range: 14-18	Com.S.	1/5
Martin et al., 2004[44]	Australia	CS	SRQ	Sexual abuse	SRQ	SI, SP, ST & SA	Lifetime & current	2,924, response rate = 85%	55.5%	M_{age} = 14	Com.S.	2/5
Miché et al., 2019[45]	Switzerland	FU	CIDI	Sexual abuse	CIDI	SA	Current	T1: 3,021, response rate = 70.9%, T3: 2,210, response rate = 73.15%	50.7%	Range: 14-24	Com.S.	6/6
Miller et al., 2014[46]	United States	FU	CI	All forms of abuse	TSC-C & YSR	SI	Current	T1: 1,354, response rate = 65.7%, T2: response rate = n/r	50%	Range: 12-18	Com.S.	2/6
Miller et al., 2017[47]	United States	FU	CTQ-EA	Emotional abuse	SITBI	SI	Lifetime	T1: 1,108, response rate = 62%, T3: response rate = 70%	n/r	M_{age} = 11.83, range: 7-18	Com.S.	3/6
Miller et al., 2017[48]	United States	FU	MINI-KID	Sexual & physical abuse	SITBI	SI & SA	Lifetime	n/r	n/r	M_{age} = 14.69, range: 12-16	MS	4/6
Molnar et al, 1998[49]	United States	CS	AHS	Sexual & physical abuse	CI	SA	Lifetime & current	775, response rate = n/r	65%	M_{age} = 17.75, range: 12-19	SY	3/5
Molnar et al, 2001[50]	United States	CS	DIS	Sexual abuse	DIS	SI & SA	Lifetime	7,132, response rate = 82.4%	50.11%	Range: 15-24	Com.S.	4/5
Mossige et al., 2014[51]	Norway	FU	SRQ	Sexual & physical abuse	SRQ	SI & SA	Lifetime & current	T2: 9,085, response rate = 77%	41.6%	Range:18-19	Com.S.	2/6
Nickel et al., 2006[52]	Germany, Austria, and Poland	FU	LSP	Sexual abuse	Patients were observed for 12 months	SI & SA	Current	T1: 288, response rate = 87.15%, T2: 83.67	0%	M_{age} = 17.65	MS	5/6
Peters et al., 2019[53]	United States	FU	K-SADS-PL	Sexual & physical abuse	K-SADS-PL	SI & SA	Current	T2:119, response rate = 86.55%	35.9%	M_{age} = 15.3	Cli.S	2/6
Plunket et al., 2001[54]	Australia	FU	Unspecified	Sexual abuse	SRQ	SI & SA	Lifetime	T1:187, response rate = 97.9%, T2: response rate: 100%	n/r	M_{age} = 17	MS	3/6

©2020 Angelakis I et al. *JAMA Network Open*.

Rabiovitch et al., 2015[55]	United States	FU	CSEQ	Sexual & physical abuse	C-SSRS	SA	Lifetime	T1:166, response rate = 90%, T2: response rate: 100%	0%	M_{age} = 15.31, range: 13-17	DA	4/6
Raleva et al., 2014[56]	Republic of Macedonia	CS	ACEQ	All forms of abuse/neglect	n/r	SA	n/r	1,414, response rate = 90.3%	41.1%	M_{age} = 19.95, range: 18-21	Com.S.	2/5
Reigstad & Kvernmo, 2017[57]	Norway	CS	SRQ	Sexual abuse	SRQ	SA	Current & lifetime	5,874, response rate = 83.1%	49.9%	Range: 15-16	Com.S.	2/5
Riggs et al., 1990[58]	United States	CS	SRQ	Sexual & physical abuse	SRQ	SA	Current & lifetime	635, response rate = 94%	48.4%	M_{age} = 15.8, range: 14-17	Com.S.	2/5
Salzinger et al., 2007[59]	United States	FU	CI	Physical abuse	YRBS	SI & SA	Current & lifetime	T1:230, response rate = 86.96%, T2: response rate: 76.5%	65%	M_{age} = 16.5	MS	5/6
Schäfer et al., 2017[60]	Brazil	CS	LHA	Sexual & physical abuse	SRQ	SI	Past 30 days	3,898, response rates = 91%	45.8%	M_{age} = 14	Com.S.	2/5
Shaunesey et al., 1993[61]	United States	CS	CI	Sexual & physical abuse	SIQ	SI & SA	Current & lifetime	117, response rate = n/r	43.59%	M_{age} = 14.5, range: 13-18	MS	1/5
Sigfusdottir et al., 2013[62]	Iceland	CS	SRQ	Sexual abuse	SRQ	SI & SA	Current & lifetime	13,560, response rate = 67%	49%	M_{age} = 17.2, range: 16-19	Com.S.	0/5
Silverman et al., 1996[63]	United States	FU	CI	Sexual & physical abuse	YASR & DIS	SI & SA	Current & lifetime	T2: 777, response rate = 48.26%	50.13%	21	Com.S.	3/5
Southwick-Bensley et al., 1999[64]	United States	CS	SRQ	Overall & sexual abuse	YRBS	SI, SP & SA	Current	21,773, response rate = 22%	47.9%	Range: 13-17	Com.S.	1/5
Stein et al., 2013[65]	Israel	CS	SCID-I/P	Sexual abuse	MAST	SA	Current & lifetime	150, response rate = 70%	0%	M_{age} = 16.35	MS	2/5
Stewart et al., 2017[66]	United States	CS	CTQ	Physical abuse	SITBI & BSSI	SI & SA	Current & lifetime	451, response rate = 88%	20.15%	M_{age} = 15.44, range: 13-18	MS	3/5
Swanston et al., 1997[67]	Australia	CS	SRC	Overall abuse	BDI & CDI	SI & SA	Current	168, response rate = 100%	13.10%	Range: 5-15	MS	1/5
Tossone et al., 2018[68]	United States	CS	CIQ	Sexual abuse	CIQ	SA	Lifetime	1,307, response rate = 82.63%	0%	M_{age} = 15.28,	JJI	2/5

										range: 10-18		
Unlu & Cakaloz, 2016[69]	Turkey	CS	SADF	Sexual abuse	n/r	SI & SA	n/r	254, response rate = 100%	0%	M_{age} = 15.05 range: 12-18	AC	3/5
van Bergen et al., 2018[70]	Netherlands	CS	YMR	Sexual abuse	YMR	SA	Lifetime	6,234, response rate = 90%	0%	Range: 14-16	EMG	2/5
Waldrop et al. 2007[71]	United States	CS	CI	Sexual & physical abuse	CI	SI & SA	Lifetime	5,367, response rate = 75%	49.76%	Range: 12-17	Com.S.	4/5
Wan et al., 2019[72]	China	CS	CTQ	All forms of abuse/neglect	SRQ	SI & SA	Current	15,278, response rate = 97%	49.8%	M_{age} = 15.4	Com.S.	2/5
Wanner et al., 2012[73]	Canada	FU	ACEQ	Sexual & physical abuse	DISC	SA	Lifetime	T1:230, response rate = 67.22%, T2: response rate: 58.87%	53%	M_{age} = 21.4, range: 19-24	Com.S.	2/6
Wilcox et al., 2017[74]	United States	FU	SLES	Sexual abuse	K-SADS	SI & SA	Lifetime	473, response rate = unspecified	51%	M_{age} = 16.9, range: 12-21	Cli.S.	3/6
Wong et al., 2009[75]	China	CS	CTS & CTSPC	Sexual & physical abuse	SRQ	SI	Lifetime	6,649, response rate = 99.1%	50.1%	Range: 12-16	Com.S.	1/5
Yen et al., 2013[76]	United States	FU	n/r	Sexual abuse	SIQ & A-LIFE	SA	Current	T2: 125, response rate = 83%	32.8%	M_{age} = 15.3	MS	3/6
Yoon et al., 2018[77]	United States	FU	ARR	All forms of abuse/neglect	YRBSS	SI	Current	T2: 584, response rate = 52.6%	44%	Range = 16-18	Com.S.	2/6
Ziaei et al., 2017[78]	Iran	CS	GSHS	Sexual abuse	SRQ	SP & SI	Current	1,531, response rate = 99.08%	47.9%	M_{age} = 16.1, range = 15-17	Com.S.	2/5
Zoroglu et al. 2003[79]	Turkey	CS	CANQ	All forms of abuse/neglect	SRQ	SA	Lifetime	862, response rate = 97.33%	38.9%	M_{age} = 15.9, range = 14-17	Com.S.	1/5

Note. AC = Abused Children; AHS = Adolescent Health Survey-Interview; A-LIFE = Adolescent Longitudinal Interval Follow-up Evaluation Interview; ACEQ = Adverse Childhood Experiences Questionnaire; ARR = Administrative record review; BDI = Beck's Depression Inventory; BSSI = Beck's Scale for Suicide Ideation; CDI = Children Depression Inventory; CIR = Client Information Records; C-SIS = Suicidal Ideation Sub-scale; C-SSAGA = Child Semi-Structured Assessment for the Genetics of Alcoholism; C-SSRS = Columbia Suicide Severity Rating Scale; CANQ = Childhood Abuse and Neglect Questionnaire; CAS = Child Abuse Survey; CCS = Mental Illness Clinical Classification Software; CEQ = Childhood Experiences Questionnaire; CI = Clinical Interview; CIDI = Composite International Diagnostic Interview; Cli.S. = Clinical Sample; CIQ = Caregiver Information Questionnaire; CMS = Childhood Maltreatment Survey; Com. S. = Community Sample; CS = Cross-Sectional; CSEQ = Childhood Sexual Experiences Questionnaire; CTQ = Childhood Trauma Questionnaire; CTS = Conflict Tactics Scale; CTS = Conflict Tactics scales; CTS-PC = Parent–Child Conflict Tactics Scale; CTSPC = Conflict Tactics Scales-Parent/Child version; CYC = Compulsory Youth Camps; DA = Delinquent Adolescents; DIS = Diagnostic Interview Schedule; DISC = Diagnostic Interview Schedule for children; EMG = Ethnic Minority Group; Forensic Interview = FI; FU = Follow-Up; GSHS = Global School-based Student Health Survey; Incar.Ind. = Incarcerated Individuals; JJI = Juvenile Justice-Involved; JO = Juvenile Offenders; K-SADS = Schedule for Affective Disorders for School-Age Children; K-SADS-PL = Schedule for Affective Disorder and Schizophrenia Interview; LHA= Life History of Abuse; LSP = Life Story & Partnership; MAST = Multi-Attitude Suicide Tendency Scale; M = Mean; Medical Charts = MC; Manual Chart Review = MCR; MINI-KID = Mini International Neuropsychiatric Interview for Children and Adolescents; MS = Mixed Sample; MSS = Minnesota Student Survey; MSSI = Modified Scale for Suicide Ideation; NAHS = Navajo Adolescent Health; NCS-A = National Comorbidity Survey and Adolescents; n/r = Not reported; Q.A = Quality appraisal of the methodology of the included studies; PCC = Psychiatric Consultation

©2020 Angelakis I et al. *JAMA Network Open*.

Checklist; PRCAS = Personal Report of Childhood Abuse Scale; R = Retrospective; RY = Runaway youth; SA = Suicide Attempts; SADF = Sexual Abuse Data Form; SBQ-R = Suicidal Behaviors Questionnaire-Revised; SCID-I/P = Structured Clinical Interview for DSM-IV Axis I Disorders/Patient Edition; SD = Standard deviation; SI = Suicidal Ideation; SIQ = Suicide Ideation Questionnaire; SITBI = Self- Injurious Thoughts and Behaviors Interview; SITBI = Self-Injurious Thoughts and Behaviors Interview; SLES = Stressful Life Events Schedule Child-Reported Version; SP = Suicide Plans; SPS = Suicide probability Scale; SR = Substantiated Reports; SRC = Self-Report Checklists; SRQ = Self-Report Questions; ST = Suicide Threats; SSB = Scale for Suicidal Behavior; SSRS = Suicide Severity Rating Scale; SY = Street Youth; TEQ-A = Traumatic Experience Questionnaire-Adolescents; TSC-C & YSR = Trauma Symptoms Checklist for Children & the Youth Self Report; VT = Victims of Trafficking; YASR = Young Adult Self-Report; YMR = Child and Adolescent Health Surveillance Monitor; YRBS = Youth Risk Behavior Survey; YRBSS = Youth Risk Behavior Surveillance System.

©2020 Angelakis I et al. *JAMA Network Open*.

eReferences.

1. Alavi N, Reshetukha T, Prost E, et al. Relationship between bullying and suicidal behaviour in youth presenting to the emergency department. *J Can Acad Child Adolesc Psychiatry*. 2017;26(2):70-77. Medline:28747929
2. Beattie TS, Prakash R, Mazzuca A, et al. Prevalence and correlates of psychological distress among 13-14 year old adolescent girls in North Karnataka, South India: a cross-sectional study. *BMC Public Health*. 2019;19(1):48. Medline:30630455 doi:10.1186/s12889-018-6355-z
3. Beautrais AL, Joyce PR, Mulder RT. Risk factors for serious suicide attempts among youths aged 13 through 24 years. *J Am Acad Child Adolesc Psychiatry*. 1996;35(9):1174-1182. Medline:8824061 doi:10.1097/00004583-199609000-00015
4. Bergen HA, Martin G, Richardson AS, Allison S, Roeger L. Sexual abuse and suicidal behavior: a model constructed from a large community sample of adolescents. *J Am Acad Child Adolesc Psychiatry*. 2003;42(11):1301-1309. Medline:14566167 doi:10.1097/01.chi.0000084831.67701.d6
5. Blasco MJVG, Vilagut G, Alayo I, et al; UNIVERSAL study group. First-onset and persistence of suicidal ideation in university students: a one-year follow-up study. *J Affect Disord*. 2019;256(256):192-204. Medline:31177047 doi:10.1016/j.jad.2019.05.035
6. Brent DA, Greenhill LL, Compton S, et al. The Treatment of Adolescent Suicide Attempters study (TASA): predictors of suicidal events in an open treatment trial. *J Am Acad Child Adolesc Psychiatry*. 2009;48(10):987-996. Medline:19730274 doi:10.1097/CHI.0b013e3181b5dbe4
7. Bruffaerts R, Demyttenaere K, Borges G, et al. Childhood adversities as risk factors for onset and persistence of suicidal behaviour. *Br J Psychiatry*. 2010;197(1):20-27. Medline:20592429 doi:10.1192/bjp.bp.109.074716
8. Cha CB, Nock MK. Emotional intelligence is a protective factor for suicidal behavior. *J Am Acad Child Adolesc Psychiatry*. 2009;48(4):422-430. Medline:19318882 doi:10.1097/CHI.0b013e3181984f44
9. Chan LF, Maniam T, Saini SM, et al. Sexual abuse and substance abuse increase risk of suicidal behavior in Malaysian youth. *Asia Pac Psychiatry*. 2013;5(suppl 1):123-126. Medline:23857848 doi:10.1111/appy.12057
10. Chen J, Dunne MP, Han P. Child sexual abuse in China: a study of adolescents in four provinces. *Child Abuse Negl*. 2004;28(11):1171-1186. Medline:15567022 doi:10.1016/j.chiabu.2004.07.003
11. Chen J, Dunne MP, Han P. Child sexual abuse in Henan province, China: associations with sadness, suicidality, and risk behaviors among adolescent girls. *J Adolesc Health*. 2006;38(5):544-549. Medline:16635765 doi:10.1016/j.jadohealth.2005.04.001
12. Cheung K, Taillieu T, Turner S, et al. Individual-level factors related to better mental health outcomes following child maltreatment among adolescents. *Child Abuse Negl*. 2018;79:192-202. Medline:29477612 doi:10.1016/j.chiabu.2018.02.007
13. Cohen Y, Spirito A, Sterling C, et al. Physical and sexual abuse and their relation to psychiatric disorder and suicidal behavior among adolescents who are psychiatrically hospitalized. *J Child Psychol Psychiatry*. 1996;37(8):989-993. Medline:9119945 doi:10.1111/j.1469-7610.1996.tb01495.x
14. Deykin EY, Buka SL. Suicidal ideation and attempts among chemically dependent adolescents. *Am J Public Health*. 1994;84(4):634-639. Medline:8154569 doi:10.2105/AJPH.84.4.634
15. Dunn EC, McLaughlin KA, Slopen N, Rosand J, Smoller JW. Developmental timing of child maltreatment and symptoms of depression and suicidal ideation in young adulthood: results from the National Longitudinal Study of Adolescent Health. *Depress Anxiety*. 2013;30(10):955-964. Medline:23592532 doi:10.1002/da.22102
16. Edgardh K, Ormstad K. Prevalence and characteristics of sexual abuse in a national sample of Swedish seventeen-year-old boys and girls. *Acta Paediatr*. 2000;89(3):310-319. Medline:10772279 doi:10.1111/j.1651-2227.2000.tb01333.x
17. Eisenberg ME, Ackard DM, Resnick MD. Protective factors and suicide risk in adolescents with a history of sexual abuse. *J Pediatr*. 2007;151(5):482-487. Medline:17961690 doi:10.1016/j.jpeds.2007.04.033
18. Esposito CL, Clum GA. Social support and problem-solving as moderators of the relationship between childhood abuse and suicidality: applications to a delinquent population. *J Trauma Stress*. 2002;15(2):137-146. Medline:12013065 doi:10.1023/A:1014860024980
19. Fried LE, Williams S, Cabral H, Hacker K. Differences in risk factors for suicide attempts among 9th and 11th grade youth: a longitudinal perspective. *J Sch Nurs*. 2013;29(2):113-122. Medline:23008186 doi:10.1177/1059840512461010
20. Garnefski N, Arends E. Sexual abuse and adolescent maladjustment: differences between male and female victims. *J Adolesc*. 1998;21(1):99-107. Medline:9503078 doi:10.1006/jado.1997.0132
21. Glowinski AL, Bucholz KK, Nelson EC, et al. Suicide attempts in an adolescent female twin sample. *J Am Acad Child Adolesc Psychiatry*. 2001;40(11):1300-1307. Medline:11699804 doi:10.1097/00004583-200111000-00010
22. Gomez SH, Tse J, Wang Y, et al. Are there sensitive periods when child maltreatment substantially elevates suicide risk? results from a nationally representative sample of adolescents. *Depress Anxiety*. 2017;34(8):734-741. Medline:28544045 doi:10.1002/da.22650
23. Grossman DC, Milligan BC, Deyo RA. Risk factors for suicide attempts among Navajo adolescents. *Am J Public Health*. 1991;81(7):870-874. Medline:2053663 doi:10.2105/AJPH.81.7.870
24. Guo L, Wang W, Gao X, Huang G, Li P, Lu C. Associations of childhood maltreatment with single and multiple suicide attempts among older Chinese adolescents. *J Pediatr*. 2018;196:244-250.e1. Medline:29526470 doi:10.1016/j.jpeds.2018.01.032
25. Handley ED, Adams TR, Manly JT, Cicchetti D, Toth SL. Mother-daughter interpersonal processes underlying the association between child maltreatment and adolescent suicide ideation. *Suicide Life Threat Behav*. 2019;49(5):1232-1240. Medline:30328155 doi:10.1111/sltb.12522
26. Harrison PA, Hoffmann NG. Sexual abuse correlates: similarities between male and female adolescents in chemical dependency treatment. *J Adolesc Res*. 1989;4(3):385-399. doi:10.1177/074355488943008
27. Haynie DL, Petts RJ, Maimon D, Piquero AR. Exposure to violence in adolescence and precocious role exits. *J Youth Adolesc*. 2009;38(3):269-286. Medline:19636744 doi:10.1007/s10964-008-9343-2
28. Hébert M, Amédée LM, Blais M, Gauthier-Duchesne A. Child sexual abuse among a representative sample of Quebec high school students: prevalence and association with mental health problems and health-risk behaviors. *Can J Psychiatry*. 2019;64(12):846-854. Medline:31299163 doi:10.1177/0706743719861387
29. Hu MH, Huang GS, Huang JL, et al. Clinical characteristic and risk factors of recurrent sexual abuse and delayed reported sexual abuse in childhood. *Medicine (Baltimore)*. 2018;97(14):e0236. Medline:29620636 doi:10.1097/MD.0000000000010236
30. Isohookana R, Riala K, Hakko H, Räsänen P. Adverse childhood experiences and suicidal behavior of adolescent psychiatric inpatients. *Eur Child Adolesc Psychiatry*. 2013;22(1):13-22. Medline:22842795 doi:10.1007/s00787-012-0311-8
31. Kaplan SJ, Pelcovitz D, Salzinger S, Mandel F, Weiner M, Labruna V. Adolescent physical abuse and risk for suicidal behaviors. *J Interpers Violence*. 1999;14(9):976-988. doi:10.1177/088626099014009005
32. Kaplan C, Tarlow N, Stewart JG, Aguirre B, Galen G, Auerbach RP. Borderline personality disorder in youth: the prospective impact of child abuse on non-suicidal self-injury and suicidality. *Compr Psychiatry*. 2016;71:86-94. Medline:27649322 doi:10.1016/j.comppsych.2016.08.016
33. Karatekin C, Almy B, Mason SM, Borowsky I, Barnes A. Mental and physical health profiles of maltreated youth. *Child Abuse Negl*. 2018;84:23-33. Medline:30036690 doi:10.1016/j.chiabu.2018.07.019

34. Kemp K, Tolou-Shams M, Conrad S, Dauria E, Neel K, Brown L. Suicidal ideation and attempts among court-involved, non-incarcerated youth. *J Forensic Psychol Pract*. 2016;16(3):169-181. Medline:29142507 doi:10.1080/15228932.2016.1172424
35. Kılıç F, Coşkun M, Bozkurt H, Kaya İ, Zoroğlu S. Self-injury and suicide attempt in relation with trauma and dissociation among adolescents with dissociative and non-dissociative disorders. *Psychiatry Investig*. 2017;14(2):172-178. Medline:28326115 doi:10.4306/pi.2017.14.2.172
36. Kiss L, Yun K, Pocock N, Zimmerman C. Exploitation, violence, and suicide risk among child and adolescent survivors of human trafficking in the greater Mekong subregion. *JAMA Pediatr*. 2015;169(9):e152278. Medline:26348864 doi:10.1001/jamapediatrics.2015.2278
37. Kurtz PD, Kurtz GL, Jarvis SV. Problems of maltreated runaway youth. *Adolescence*. 1991;26(103):543-555. Medline:1962537
38. Kwok SYCL, Yeung JWK, Low AYT, Lo HHM, Tam CHL. The roles of emotional competence and social problem-solving in the relationship between physical abuse and adolescent suicidal ideation in China. Child Abuse Negl. 2015;44:117-129.
39. Li X, You J, Ren Y, et al. A longitudinal study testing the role of psychache in the association between emotional abuse and suicidal ideation. *J Clin Psychol*. 2019;75(12):2284-2292. Medline:31468529 doi:10.1002/jclp.22847
40. Lipschitz DS, Winegar RK, Nicolaou AL, Hartnick E, Wolfson M, Southwick SM. Perceived abuse and neglect as risk factors for suicidal behavior in adolescent inpatients. *J Nerv Ment Dis*. 1999;187(1):32-39. Medline:9952251 doi:10.1097/00005053-199901000-00006
41. Lynskey MT, Fergusson DM. Factors protecting against the development of adjustment difficulties in young adults exposed to childhood sexual abuse. *Child Abuse Negl*. 1997;21(12):1177-1190. Medline:9429770 doi:10.1016/S0145-2134(97)00093-8
42. Lyon ME, Benoit M, O'Donnell RM, Getson PR, Silber T, Walsh T. Assessing African American adolescents' risk for suicide attempts: attachment theory. *Adolescence*. 2000;35(137):121-134. Medline:10841301
43. Martin G. Reported family dynamics, sexual abuse, and suicidal behaviors in community adolescents. *Arch Suicide Res*. 1996;2(3):183-195. doi:10.1080/13811119608259000
44. Martin G, Bergen HA, Richardson AS, Roeger L, Allison S. Sexual abuse and suicidality: gender differences in a large community sample of adolescents. *Child Abuse Negl*. 2004;28(5):491-503. Medline:15159067 doi:10.1016/j.chiabu.2003.08.006
45. Miche M, Hofer PD, Voss C, et al. Specific traumatic events elevate the risk of a suicide attempt in a 10-year longitudinal community study on adolescents and young adults. *Eur Child Adolesc Psychiatry*. 2020;29(2):179-186. Medline:31054127
46. Miller AB, Adams LM, Esposito-Smythers C, Thompson R, Proctor LJ. Parents and friendships: a longitudinal examination of interpersonal mediators of the relationship between child maltreatment and suicidal ideation. *Psychiatry Res*. 2014;220(3):998-1006. Medline:25454119 doi:10.1016/j.psychres.2014.10.009
47. Miller AB, Jenness JL, Oppenheimer CW, Gottleib AL, Young JF, Hankin BL. Childhood emotional maltreatment as a robust predictor of suicidal ideation: a 3-year multi-wave, prospective investigation. *J Abnorm Child Psychol*. 2017;45(1):105-116. Medline:27032784 doi:10.1007/s10802-016-0150-z
48. Miller AB, Eisenlohr-Moul T, Giletta M, et al. A within-person approach to risk for suicidal ideation and suicidal behavior: examining the roles of depression, stress, and abuse exposure. *J Consult Clin Psychol*. 2017;85(7):712-722. Medline:28425734 doi:10.1037/ccp0000210
49. Molnar BE, Shade SB, Kral AH, Booth RE, Watters JK. Suicidal behavior and sexual/physical abuse among street youth. *Child Abuse Negl*. 1998;22(3):213-222. Medline:9589175 doi:10.1016/S0145-2134(97)00137-3
50. Molnar BE, Berkman LF, Buka SL. Psychopathology, childhood sexual abuse and other childhood adversities: relative links to subsequent suicidal behaviour in the US. *Psychol Med*. 2001;31(6):965-977. Medline:11513382 doi:10.1017/S0033291701004329
51. Mossige S, Huang L, Straiton M, Katrina R. Suicidal ideation and self-harm among youths in Norway: associations with verbal, physical and sexual abuse. *Child Fam Soc Work*. 2014;21(2):166-175. doi:10.1111/cfs.12126
52. Nickel C, Simek M, Moleda A, et al. Suicide attempts versus suicidal ideation in bulimic female adolescents. *Pediatr Int*. 2006;48(4):374-381. Medline:16911082 doi:10.1111/j.1442-200X.2006.02224.x
53. Peters JR, Mereish EH, Solomon JB, Spirito AS, Yen S. Suicide ideation in adolescents following inpatient hospitalization: examination of intensity and lability over 6 months. *Suicide Life Threat Behav*. 2019;49(2):572-585. Medline:29577366 doi:10.1111/sltb.12448
54. Plunkett A, O'Toole B, Swanston H, Oates RK, Shrimpton S, Parkinson P. Suicide risk following child sexual abuse. *Ambul Pediatr*. 2001;1(5):262-266. Medline:11888413 doi:10.1367/1539-4409(2001)001<0262:SRFCSA>2.0.CO;2
55. Rabinovitch SM, Kerr DC, Leve LD, Chamberlain P. Suicidal behavior outcomes of childhood sexual abuse: longitudinal study of adjudicated girls. *Suicide Life Threat Behav*. 2015;45(4):431-447. Medline:25370436 doi:10.1111/sltb.12141
56. Raleva M, Jordanova Peshevska D, Filov I, et al. Childhood abuse, household dysfunction and the risk of attempting suicide in a national sample of secondary school and university students. *Open Access Maced J Med Sci*. 2014;2(2):379-383. doi:10.3889/oamjms.2014.065
57. Reigstad B, Kvernmo S. Concurrent adversities and suicide attempts among Sami and non-Sami adolescents: the Norwegian Arctic Adolescent Study (NAAHS). *Nord J Psychiatry*. 2017;71(6):425-432. Medline:28486095 doi:10.1080/08039488.2017.1315175
58. Riggs S, Alario AJ, McHorney C. Health risk behaviors and attempted suicide in adolescents who report prior maltreatment. *J Pediatr*. 1990;116(5):815-821. Medline:2329431 doi:10.1016/S0022-3476(05)82679-4
59. Salzinger S, Rosario M, Feldman RS, Ng-Mak DS. Adolescent suicidal behavior: associations with preadolescent physical abuse and selected risk and protective factors. *J Am Acad Child Adolesc Psychiatry*. 2007;46(7):859-866. Medline:17581450 doi:10.1097/chi.0b013e318054e702
60. Schäfer JL, Teixeira VA, da Fontoura LP, de Castro LC, Horta RL. Exposure to physical and sexual violence and suicidal ideation among schoolchildren. *Br J Psychiatry*. 2017;66(2):96-103. Medline:27908895 doi:10.1590/0047-2085000000156
61. Shaunesey K, Cohen JL, Plummer B, Berman A. Suicidality in hospitalized adolescents: relationship to prior abuse. *Am J Orthopsychiatry*. 1993;63(1):113-119. Medline:8427301 doi:10.1037/h0079411
62. Sigfusdottir ID, Asgeirsdottir BB, Gudjonsson GH, Sigurdsson JF. Suicidal ideations and attempts among adolescents subjected to childhood sexual abuse and family conflict/violence: the mediating role of anger and depressed mood. *J Adolesc*. 2013;36(6):1227-1236. Medline:24215969 doi:10.1016/j.adolescence.2013.10.001
63. Silverman AB, Reinherz HZ, Giaconia RM. The long-term sequelae of child and adolescent abuse: a longitudinal community study. *Child Abuse Negl*. 1996;20(8):709-723. Medline:8866117 doi:10.1016/0145-2134(96)00059-2
64. Bensley LS, Van Eenwyk J, Spieker SJ, Schoder J. Self-reported abuse history and adolescent problem behaviors, I: antisocial and suicidal behaviors. *J Adolesc Health*. 1999;24(3):163-172. Medline:10195799 doi:10.1016/S1054-139X(98)00111-6
65. Stein D, Zinman D, Halevy L, et al. Attitudes toward life and death and suicidality among inpatient female adolescents with eating disorders. *J Nerv Ment Dis*. 2013;201(12):1066-1071. Medline:24284642 doi:10.1097/NMD.0000000000000055
66. Stewart JG, Esposito EC, Glenn CR, et al. Adolescent self-injurers: comparing non-ideators, suicide ideators, and suicide attempters. *J Psychiatr Res*. 2017;84:105-112. Medline:27716512 doi:10.1016/j.jpsychires.2016.09.031
67. Swanston HY, Tebbutt JS, O'Toole BI, Oates RK. Sexually abused children 5 years after presentation: a case-control study. Pediatrics. 1997;100(4):600-608. doi:10.1542/peds.100.4.600

©2020 Angelakis I et al. *JAMA Network Open*.

68. Tossone K, Wheeler M, Butcher F, Kretschmar J. The role of sexual abuse in trauma symptoms, delinquent and suicidal behaviors, and criminal justice outcomes among females in a juvenile justice diversion program. Violence Against Women. 2018;24(8):973-993. doi:10.1177/1077801217724921
69. Unlu G, Cakaloz B. Effects of perpetrator identity on suicidality and nonsuicidal self-injury in sexually victimized female adolescents. Neuropsychiatr Dis Treat. 2016;12:1489-1497. doi:10.2147/NDT.S109768
70. van Bergen DD, Eikelenboom M, van de Looij-Jansen PP. Attempted suicide of ethnic minority girls with a Caribbean and Cape Verdean background: rates and risk factors. BMC Psychiatry. 2018;18(1):14. doi:10.1186/s12888-017-1585-7
71. Waldrop AE, Hanson RF, Resnick HS, Kilpatrick DG, Naugle AE, Saunders BE. Risk factors for suicidal behavior among a national sample of adolescents: implications for prevention. J Trauma Stress. 2007;20(5):869-879. doi:10.1002/jts.20291
72. Wan Y, Chen R, Ma S, et al. Associations of adverse childhood experiences and social support with self-injurious behaviour and suicidality in adolescents. Br J Psychiatry. 2019;214(3):146-152. doi:10.1192/bjp.2018.263
73. Wanner B, Vitaro F, Tremblay RE, Turecki G. Childhood trajectories of anxiousness and disruptiveness explain the association between early-life adversity and attempted suicide. Psychol Med. 2012;42(11):2373-2382. doi:10.1017/S0033291712000438
74. Wilcox HC, Fullerton JM, Glowinski AL, et al. Traumatic stress interacts with bipolar disorder genetic risk to increase risk for suicide attempts. J Am Acad Child Adolesc Psychiatry. 2017;56(12):1073-1080. doi:10.1016/j.jaac.2017.09.428
75. Wong WC, Leung PW, Tang CS, Chen WQ, Lee A, Ling DC. To unfold a hidden epidemic: prevalence of child maltreatment and its health implications among high school students in Guangzhou, China. Child Abuse Negl. 2009;33(7):441-450. doi:10.1016/j.chiabu.2008.02.010
76. Yen S, Weinstock LM, Andover MS, Sheets ES, Selby EA, Spirito A. Prospective predictors of adolescent suicidality: 6-month post-hospitalization follow-up. Psychol Med. 2013;43(5):983-993. doi:10.1017/S0033291712001912
77. Yoon Y, Cederbaum JA, Schwartz A. Childhood sexual abuse and current suicidal ideation among adolescents: Problem-focused and emotion-focused coping skills. J Adolesc. 2018;67:120-128. doi:10.1016/j.adolescence.2018.06.009
78. Ziaei R, Viitasara E, Soares J, et al. Suicidal ideation and its correlates among high school students in Iran: a cross-sectional study. BMC Psychiatry. 2017;17(1):147. doi:10.1186/s12888-017-1298-y
79. Zoroglu SS, Tuzun U, Sar V, et al. Suicide attempt and self-mutilation among Turkish high school students in relation with abuse, neglect and dissociation. Psychiatry Clin Neurosci. 2003;57(1):119-126. doi:10.1046/j.1440-1819.2003.01088.x

©2020 Angelakis I et al. *JAMA Network Open*.
